# Fallopian tube lavage sampling towards early detection of pre‐invasive ovarian cancer

**DOI:** 10.1002/ctm2.70557

**Published:** 2026-01-02

**Authors:** Melanie Seaton, Sarah Harbach, Joanne Oke, Thomas D. J. Walker, Jessica Dalton‐O'Reilly, Julian Selley, Daniel R. Brison, James Bolton, Stefan Meyer, David Knight, Richard J. Edmondson, Christine K. Schmidt

**Affiliations:** ^1^ Manchester Cancer Research Centre Division of Cancer Sciences Faculty of Biology School of Medical Sciences Medicine and Health University of Manchester Manchester UK; ^2^ Department of Gynaecological Oncology Division of Cancer Sciences Faculty of Biology School of Medical Sciences Medicine and Health University of Manchester Manchester UK; ^3^ Biological Mass Spectrometry Core Facility Faculty of Biology Medicine and Health Manchester Academic Health Science Centre University of Manchester Manchester UK; ^4^ Maternal and Fetal Health Research Centre Division of Developmental Biology and Medicine Faculty of Biology Medicine and Health School of Medical Sciences University of Manchester Manchester UK; ^5^ Department of Reproductive Medicine St Mary's Hospital, Manchester University NHS Foundation Trust Manchester Academic Health Sciences Centre Manchester UK; ^6^ Department of Pathology Manchester University NHS Foundation Trust Manchester Academic Health Science Centre Manchester UK; ^7^ Department of Paediatric Haematology and Oncology Royal Manchester Children's Hospital Manchester UK; ^8^ Young Oncology Unit The Christie NHS Foundation Trust Manchester UK; ^9^ St Mary's Hospital Central Manchester NHS Foundation Trust Manchester Academic Health Science Centre Manchester UK

1

Dear Editor,

Most high‐grade serous ovarian cancer (HGSOC) cases arise from pre‐invasive fallopian tube (FT) lesions, known as serous tubal intraepithelial carcinomas (STICs). Yet, despite an estimated 6‐ to 7‐year window‐of‐opportunity, no clinical test exists for detecting these at an early, treatable stage.^1^ To address this, we developed and applied a novel sampling technique to obtain FT lavage, enabling analysis of early tumour‐associated molecular changes in FT‐derived biofluids.

To establish the feasibility of this technique, we collected 27 FT lavages from high‐risk *BRCA1/2* mutation carriers, ovarian neoplasm (ON) patients, and control individuals with benign or non‐tubal gynaecological conditions (Figure 1A and B; Supplementary Discussions 1 and 2). Lavages were acquired from FTs directly after surgical removal by inserting a PBS‐filled syringe into the FT lumen beyond the fimbrial end and flushing towards the utero‐tubal junction (Figure 2A). Sampling away from the fimbriae enabled us to test for signals diffusing from any lesions proximally along the tube, where they would be accessible via minimally invasive trans‐cervical falloposcopy,^2^ supporting potential clinical feasibility (Supplementary Discussion 3).

**FIGURE 1:**
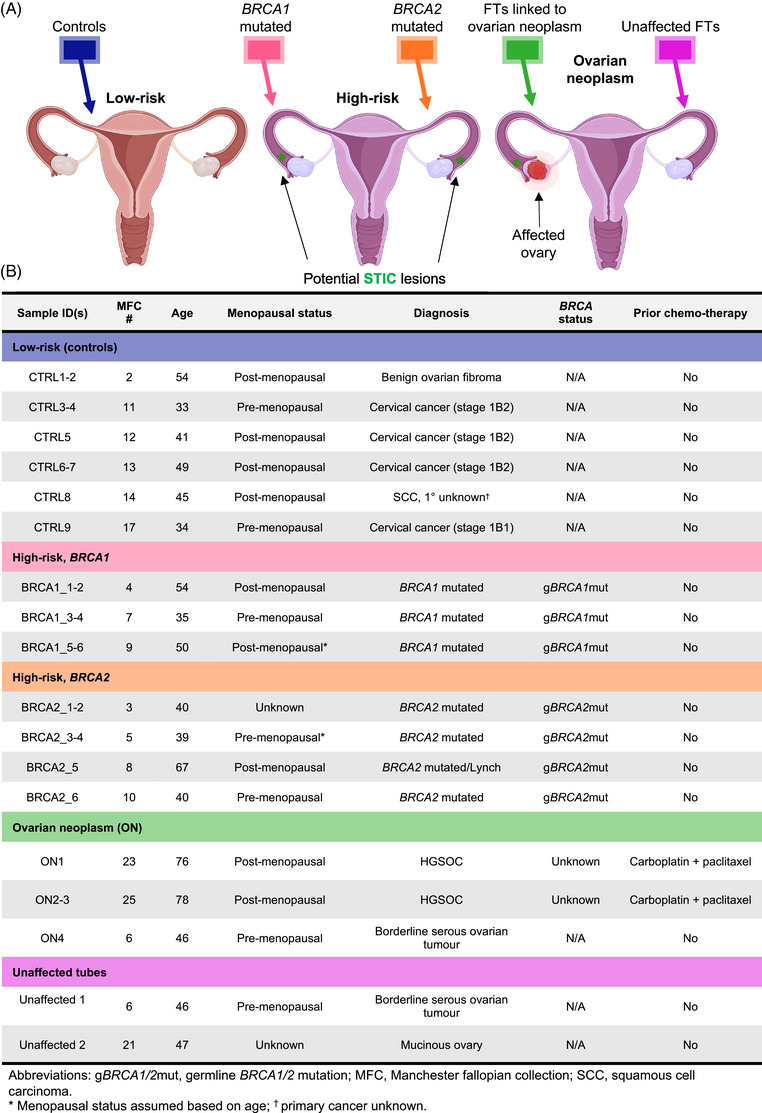
Patient stratification and clinical information of sampled fallopian tube lavages. (A) Schematic, illustrating the different patient groups from whom fallopian tube (FT) lavage samples were collected: women having surgery for cervical cancer, squamous cell carcinoma (SCC) or benign ovarian fibroma without ovarian cancer or known mutations in *BRCA1*, *BRCA2* or other homologous recombination genes (group 1: low‐risk/controls; blue), women at high risk of developing ovarian cancer (groups 2/3: *BRCA1/2*‐mutation carriers; salmon/orange), patients diagnosed with an ovarian neoplasm, with affected (group 4: green) and unaffected (group 5: purple) FTs. Created with BioRender.com. (B) Patient characteristics and clinical information for FT lavage samples used in the study.

**FIGURE 2:**
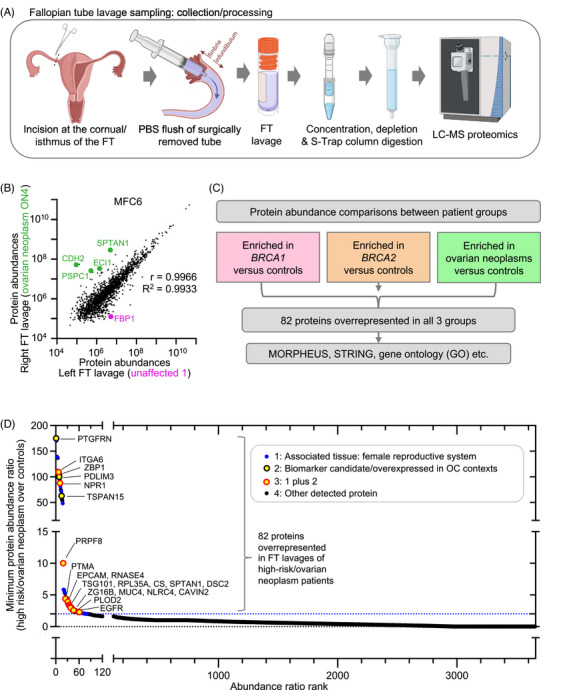
Candidate protein signatures delineating fallopian tube lavages of high‐risk/ovarian neoplasm patients. (A) Schematic illustrating the collection and processing steps of fallopian tube (FT) samples for analysis, including their concentration, depletion of common blood proteins and S‐trap column digestion before analysis by liquid chromatography mass spectrometry (LC‐MS). FT lavage samples were obtained by inserting a syringe containing PBS into the fimbrial end, with the syringe tip positioned just beyond the fimbrial ostium within the tubal lumen. Sterile PBS was gently introduced, without applying pressure, towards the utero‐tubal junction, and the resulting fluid was collected for downstream analysis. Created using BioRender.com. (B) Dot plot depicting the correlation of detected proteins obtained for the left and right tube of a patient diagnosed with a unilateral ovarian neoplasm (MFC6, see Figure 1B for patient information). Proteins of interest, notably more abundant in the neoplasm or unaffected tube, are highlighted in green and pink, respectively. Note that the comparison is based on *n* = 1 patient with no statistical significance implied. (C) Flow chart showing analytical pipeline from raw data through to altered protein signatures. After removal of blood proteins from raw data, protein abundances were compared between different risk groups/ovarian neoplasm patients, which identified 82 over‐represented proteins in *BRCA*‐mutation carriers and ovarian neoplasm patients. (D) Plot highlighting the 82 proteins overrepresented in high‐risk/ovarian neoplasm FT lavages compared to controls. Colour‐coding indicates association of proteins with the female reproductive tract (identified using STRING, blue circles), proteins with literature backing as OC biomarker candidates or overexpressed in OC context (yellow circles, black outline), or both (yellow circles, red outline). Note the large number of identified candidates showing tissue‐of‐origin relevance and/or involvement in ovarian cancer‐related pathways, underscoring the biological plausibility of the observed changes; the threshold of 2 is indicated as a blue dotted line. FT: fallopian tube; HGSOC: high‐grade serous ovarian cancer; LC‐MS: liquid chromatography‐mass spectrometry; OC: ovarian cancer.

As FTs were flushed post‐surgical removal, effects of blood contamination on downstream analysis were reduced through selective depletion of common blood proteins during sample preparation (Figure 2A), and removal of abundant plasma proteins during analysis (Figure S1). Proteomic analysis revealed > 2000 proteins per sample with significant overlap between samples (Figure S1a and b, Table S1), including FT‐resident mucins (MUC16/CA125, MUC5B, MUC5AC) and oviduct‐specific glycoprotein 1 (OVGP1). No major differences in the numbers of the top 400 plasma proteins detected were identified between samples (mean ± SD = 316 ± 13.2), and this was proportionally slight (8.7%) compared to overall protein numbers (Figure S1a). Intra‐patient comparison showed strong correlation between left and right FT samples for most pairs (*R* ^2 ^>0.9, Figure S2a–d), overall indicating FT lavage as a promising resource for comparative proteomic analysis across risk and disease states.

To assess the ability of FT lavage to distinguish between neoplastic and non‐neoplastic samples, we compared proteomes of a left and right FT lavage from a patient with a unilateral ovarian neoplasm (MFC6), thereby controlling for inter‐patient variability and confounding factors. Despite high overall correlation (*R* ^2 ^= 0.9933), several tumour‐promoting proteins were enriched in the neoplasm‐associated FT, such as SPTAN1, which is overexpressed in early OC lesions in the FT,^3^ while tumour suppressor fructose‐1,6‐bisphosphatase 1 (FBP1)^4^ was reduced (Figure 2B, Table S2), suggesting that our workflow has potential to identify proteins in FT lavage linked to tumour progression and local neoplastic changes.

We next hypothesised that a subset of the high‐risk group may already exhibit early molecular changes associated with disease progression, detectable by comparison to cancer‐associated FT profiles. We therefore compared FT lavage proteomes from high‐risk and ovarian neoplasm groups to low‐risk controls (Figure 2C). Supporting our hypothesis, 82 proteins were commonly elevated more than or equal to twofold across the *BRCA1*/*2*‐mutated and ovarian neoplasm groups over controls (Figure 2D, Tables S1 and S2). A marked proportion were representative of the female reproductive system and, in addition to identifying proteins not previously associated with OC, we detected others with established roles in OC progression, metastasis, and prognosis (Figure 2D, Table S2). Key proteins included PTGFRN, detected more frequently in plasma from OC patients than controls,^5^ ITGA6, linked to increased proliferation and metastasis of OC cells,^6^ and ZBP1, a reported necroptosis mediator in OC^7^ with overexpression of its enhancer regions in *BRCA1*‐mutated cells, consistent with ZBP1 enrichment in our *BRCA*‐mutant FT lavages.^8^ Notably, several proposed biomarkers for advanced disease stages and OC drug targets were detected, highlighting potential relevance to early detection. Cellular component enrichment highlighted extracellular and cell membrane proteins as predominant gene ontologies, alongside extracellular vesicles (Figure S3a), suggesting increased secretory activity as a potential source of these proteins.

Functional protein network and clustering analysis revealed networks associated with actin, apoptosis, cilia, and signalling (Figure S3b). A central EGFR cluster linked to ITGA6, EPCAM and others involved in cancer pathways, including MAPK and PI3K‐AKT (Table S2). Other clusters included proteins linked to carcinogenesis and the FT epithelium. Hierarchical clustering of the different risk/ovarian neoplasm groups revealed that *BRCA1/2*‐mutated groups showed similar protein profiles (Figure S3c). Additionally, controls clustered closely with unaffected FTs from women with unilateral tumours. Notably, the latter were not used to define the protein signature, underscoring our approach's potential for cancer early detection.

Further hierarchical clustering of individual lavages revealed two main clusters, with marked separation of control and most *BRCA*‐mutated samples from ovarian neoplasms, reflecting expected molecular similarity between average and most high‐risk FTs (Figure 3A). However, in one *BRCA1*‐mutated individual, lavages from their FTs clustered separately, with *BRCA1_2* clustering more closely with an HGSOC sample (Figure 3A). To determine whether this aberrant molecular profile correlated with histological changes, we examined further H&E sections from the FFPE blocks of the fimbrial ends of both FTs. This revealed a STIC lesion in one FT with typical morphology, aberrant p53 expression and increased Ki67 proliferation index on immunohistochemistry (Figure 3B). The lesion had been previously undetected on initial standard sectioning and by entirely sampling the fimbrial ends according to the SEE‐FIM protocol. Although one instance is not sufficient for firm conclusions, considering the rarity of further STIC lesion detection upon deeper sectioning,^9^ this is unlikely to be by chance (Supplementary Discussion 4).

**FIGURE 3:**
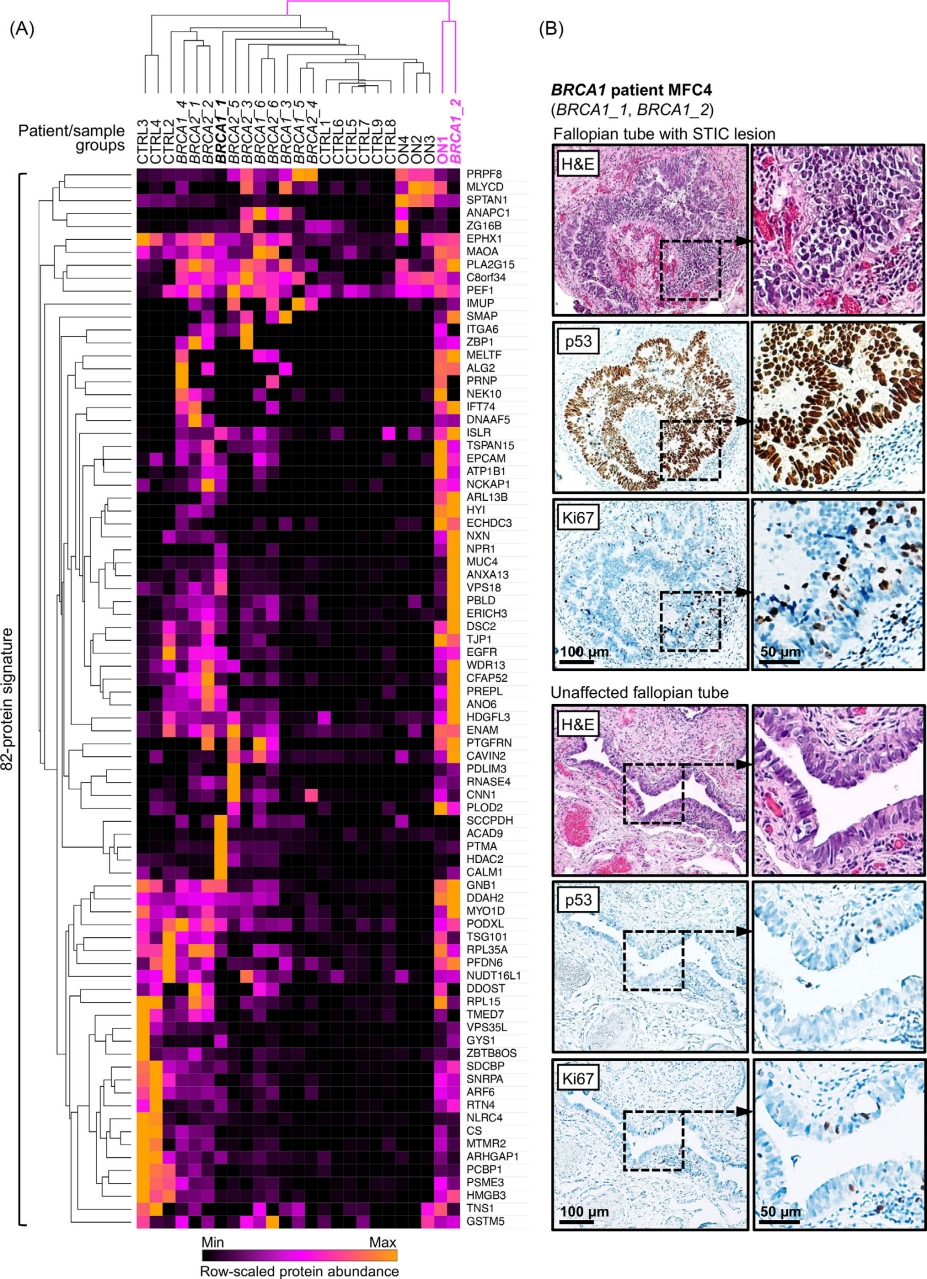
Hierarchical clustering of 82‐protein candidate signature for individual patients and retrospective identification of an incidental STIC. (A) Heatmap and hierarchical clustering of 82 overrepresented proteins for individual fallopian tube (FT) lavages from control (CTRL1‐9), high‐risk (*BRCA1_1‐6*, *BRCA2_1‐6*) and ovarian neoplasm (ON) patients (ON1‐4). Note divergent clustering of patient MFC4's two FT samples (*BRCA1_1‐2*, highlighted in bold), with one of the samples (BRCA1‐2) clustering together with an ovarian neoplasm sample (ON1) in a separate cluster, highlighted in pink. Schematic generated using Morpheus (https://software.broadinstitute.org/morpheus), with row‐scaled abundances indicated. (B) Previously unidentified serous tubal intraepithelial carcinoma (STIC) in an FT from *BRCA1* patient MFC4 (top panels) compared to normal FT epithelium in opposing FT (bottom panels). Three consecutive 4 µm sections were taken from formalin‐fixed paraffin‐embedded (FFPE) blocks previously processed with the sectioning and extensively examining the fimbriated end (SEE‐FIM) protocol, stained with H&E, or for p53 or Ki67. Scale bars indicate 100 µm (left column) or 50 µm for insets (right column). FFPE: formalin‐fixed paraffin‐embedded; FT: fallopian tube; SEE‐FIM: sectioning and extensively examining the fimbrial end; STIC: serous tubal intraepithelial carcinoma.

To validate our findings and further determine their relevance to STIC identification, we performed immunofluorescence staining of five STIC lesions from an independent cohort of three patients (Figure 4A and B). ITGA6, EGFR and EPCAM were chosen from our 82‐protein candidate signature for their centrality to our STRING network and predicted surface presence for future therapeutic targeting. ITGA6 showed consistent increased staining in STICs compared to neighbouring healthy FT epithelium (FTE), while EPCAM exhibited less pronounced alterations (Figure 4B and C). Despite variation in EGFR intensity differences, strong apical staining in healthy FTE contrasted with diffuse staining in STICs (Figure 4D and E), reflecting polarity loss^10^ and suggesting distinct mechanisms of protein accumulation in FT fluid.

**FIGURE 4:**
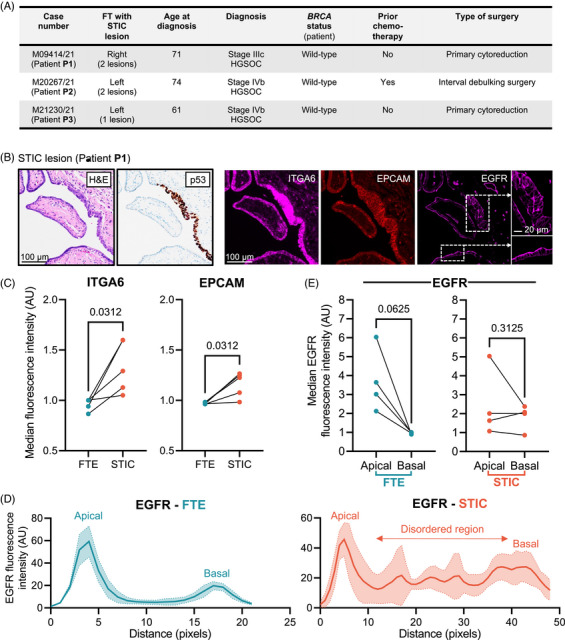
Candidate protein changes in STIC lesions from an independent patient cohort. (A) Patient characteristics and clinical information for STIC lesion‐containing fallopian tube (FT) tissue sections used in this study. Number of STICs indicates lesions analysed by immunofluorescence (IF)/immunohistochemistry (IHC). (B) IHC and IF staining of representative STIC lesions. Left panels show the characteristic H&E (left) and p53 (right) staining of a STIC lesion from patient **P1** (see (a) for patient information). Right panels depict IF staining of consecutive sections from the same formalin‐fixed paraffin‐embedded (FFPE) block for ITGA6 (left), EPCAM (middle) and EGFR (right), showing levels of the key proteins identified as overrepresented in high‐risk/ovarian neoplasm groups in a STIC lesion compared to the surrounding normal‐appearing FT epithelium (FTE). For EGFR, the concentrated staining at the apical membrane of normal‐appearing epithelium is lost in the STIC lesion (see insets). Scale bars indicate 100 µm, or 20 µm for EGFR insets. (C) Quantification of median IF staining intensities of ITGA6 and EPCAM measured across multiple regions of the STIC lesion featured in (B) and adjacent normal‐appearing FTE for two lesions for patients P1 and P2 and one lesion for patient P3 (see (A) for patient specifics). >300 points were analysed per STIC/adjacent normal FT epithelium (FTE), corrected for background and normalised to the average normal FTE fluorescence. Median fluorescence intensities were calculated for normal FTE and STIC lesions per lesion (*n* = 5). Statistical significance was determined using one‐tailed Wilcoxon matched‐pairs signed rank testing. (D) Quantification of EGFR IF in one representative lesion from patient P1 shown in (B), measured across five regions from the apical to basal axis of the STIC lesion, and across five regions of the surrounding normal‐appearing FTE. Average fluorescence intensity is plotted (solid blue/orange lines) with standard deviation indicated (dotted blue/orange lines with shading). Apical and basal regions for normal FTE and STIC lesions, alongside disordered region in STIC lesions are indicated. (E) Quantification of EGFR IF shown in (B) along multiple regions of the basal and apical membranes of the STIC lesion and normal‐appearing surrounding FTE. Two STICs were analysed for patient P1, and one STIC for patients P2 and P3 (see (A) for patient information). >300 points were analysed per STIC/adjacent normal FTE for both apical and basal membranes, corrected for background and normalised to the average normal FTE basal fluorescence. Median fluorescence intensities were calculated for apical and basal regions across normal FTE and STIC lesions; statistical significance was determined with a one‐tailed Wilcoxon matched‐pairs signed rank test. AU: arbitrary units; BRCA: breast cancer susceptibility gene; EPCAM: epithelial cell adhesion molecule; EGFR: epidermal growth factor receptor; FFPE: formalin‐fixed paraffin‐embedded; FT: fallopian tube; FTE: fallopian tube epithelium, H&E: haematoxylin & eosin; IF: immunofluorescence; IHC: immunohistochemistry; ITGA6: integrin alpha‐6; STIC: serous tubal intraepithelial carcinoma.

In conclusion, we have identified a candidate signature present in the lavage of FTs which enabled retrospective identification of a STIC lesion in a high‐risk *BRCA* mutation carrier. While further exploration and validation in larger cohorts is needed (Supplementary Discussion 5), our findings point to a promising direction for less invasive OC risk management strategies that could help delay or eliminate the need for invasive prophylactic surgeries while preserving fertility in high‐risk women.

## AUTHOR CONTRIBUTIONS

The project was conceived and supervised by CKS and RJE. MS and SH carried out the experiments and performed data analysis and interpretation, with support from JO, TDJW and JDO'R. JS and DK conducted the mass spectrometry workflows. JB provided pathological expertise and performed retrospective STIC assessments. SM and DRB contributed critical insights into relevant clinical contexts. All authors reviewed and discussed the findings and provided feedback on the manuscript.

## FUNDING

The authors acknowledge funding by a BBSRC David Phillips Fellowship (BB/N019997/1 to CKS), an MRC research grant (MR/X008754/1 to CKS), a CRUK International Alliance for Cancer Early Detection (ACED) programme grant (Novel Early Markers for Ovarian cancer (NEMO) – ACEPGM‐2023/100001), a Manchester Academic Health Science Centre Women's and Children's Domain Advanced Technologies Pump‐Priming Award, as well as MCRC/CRUK and CRUK MB PhD studentships to MS and JO.

## CONFLICT OF INTEREST STATEMENT

The authors declare no conflicts of interest.

## ETHICS STATEMENT

Fallopian tube lavages and formalin‐fixed paraffin‐embedded fallopian tube tissues were obtained via the Manchester University NHS Foundation Trust (MFT) Biobank under Human Tissue Authority (HTA)‐approved ethics applications (REC14/NW/1260 and REC19/NW/0644). All patients provided their written informed consent and were prospectively included in the study.

## Supporting information



Supporting Information.

Supporting Information.

Supporting Information.

Supporting Information.

Supporting Information.

## Data Availability

Details of the approaches and techniques used are outlined in the Supplementary Methods section. Fallopian tube lavage mass spectrometry proteomics data are available in Table S1. The raw data are accessible through the PRIDE repository (accession number: PXD072299).
