# Supplementary material for: Fallopian tube lavage sampling towards early detection of pre‐invasive ovarian cancer
Source: Clin Transl Med. 2026 Jan 2;16(1):e70557. doi: 10.1002/ctm2.70557 (PMC12759042; doi:10.1002/ctm2.70557)
Supplement: Supplementary file 1 — Supporting Information. [file CTM2-16-e70557-s005.pdf]

## SUPPLEMENTARY DISCUSSION

### 1. Choice of low-risk fallopian tube control samples

Given that access to fallopian tube lavage material is restricted to patients undergoing gynaecological surgery, truly healthy controls are rarely available in this setting. Nonetheless, several clinical scenarios provide suitable low-risk comparators for HGSOC development, including benign conditions such as uterine leiomyomatas, fibromas, as well as certain non-tubal malignancies. In this context, selected cervical cancer cases represent pragmatic controls, particularly those at stages IB1-IB2, as tubal involvement in localised disease at these stages – especially in squamous cell carcinoma of the cervix – is exceedingly rare[1]. Clinical staging and histopathological assessment can reliably confirm confinement of disease to the cervix, which was the case for all cervical cancer controls included in this feasibility study. Given that cervical cancer and serous tubo-ovarian neoplasms arise from distinct epithelial lineages and follow largely independent pathogenetic pathways[2–7], we do not anticipate that the presence of a localised cervical malignancy would obscure fallopian tube-specific proteomic alterations. However, at this early stage, we cannot fully exclude the possibility that pelvic malignancies exert systemic or paracrine effects that influence the local proteomic milieu. This will be an important aspect to investigate in larger, future cohorts incorporating additional fallopian tube samples from non-cancer patients (see also section "5. Future stratified cohorts" below).

### 2. Fallopian tube-based sampling for ovarian cancer histotypes beyond HGSOC

While the focus of this study was on HGSOC, for which a fallopian tube origin is now well established[3,4,8–11], several other ovarian cancer histotypes have been implicated to arise from the fallopian tube, including serous borderline ovarian tumours[4,12–15]. In line with this, we included a patient with a serous borderline ovarian tumour for whom fallopian tube lavages were available from both the affected and the contralateral unaffected tube. This provided a unique opportunity to assess our lavage approach in an isogenic background, without the confounding effects of inter-patient heterogeneity, which can be particularly limiting in a pilot feasibility dataset. It also gave us a first opportunity to evaluate whether local proteomic alterations in the tubal lumen may point towards neoplastic tubal changes associated with borderline tumours. In this context it is noteworthy that because the molecular pathways underpinning serous borderline tumours differ from those driving HGSOC[16,17], any such alterations – if present – would likely give rise to distinct biomarker profiles. Extending this approach to additional cancers of potential tubal origin, particularly in cases where lesions can be confirmed within the fallopian tube, may thus provide a broader perspective on the utility, sensitivity and specificity of fallopian tube-based sampling strategies. Such comparisons could help inform the potential applicability of tubal lavage for detecting early neoplastic transformation across different tubal-derived histotypes (see also section "5. Future stratified cohorts" below).

### 3. Next-generation falloposcopy for early detection of pre-invasive HGSOC lesions

Falloposcopy is an endoscopic technique originally developed for minimally invasive management of fallopian tube pathologies associated with infertility[18–22]. Recent advances demonstrate that *in vivo* access to the proximal fallopian tube via a trans-cervical route is feasible and can be performed safely in patients, as shown by Rocha et al., 2025[23], who reported no epithelial damage in the majority of cases and no injury to underlying tissues. These developments highlight the potential of falloposcopy as a platform for future early detection strategies aimed at identifying pre-invasive tubal lesions[24,25].

In this context, we envision next-generation falloposcopic approaches that access the tube through the uterotubal junction and sample or interrogate the proximal tubal lumen – rather than the fimbrial end – thereby avoiding manipulation of the distal fimbriae where STIC lesions typically arise. Such approaches could involve gentle swabbing or brushing of the proximal tubal

epithelium or, alternatively, deployment of emerging biosensing technologies[26,27] capable of detecting molecular signals *in vivo*. These strategies would not require applying pressure or fluid flow across the fimbrial surface and would therefore minimise any risk of dislodging epithelial cells or disseminating transformed cells.

A proximal, trans-cervical approach may also substantially reduce blood contamination compared with *ex vivo* lavage of surgically removed fallopian tubes, as no surgical incisions are involved and sampling can be performed under visually controlled conditions. Moreover, based on our findings highlighting the potential for STIC-associated signals to diffuse proximally along the tubal lumen, targeted sampling of the proximal tube may offer an accessible window for detecting early neoplastic changes even when lesions are confined to the distal fimbriae.

To fully realise the translational potential of next-generation falloposcopy for early detection of pre-invasive HGSOC and possibly other tubal diseases (see "2. Fallopian tube-based sampling for ovarian cancer histotypes beyond HGSOC" section above), further dedicated studies will be required. Such work will have to assess procedural safety, epithelial integrity, reproducibility, and feasibility across diverse patient groups, as well as determine whether proximal sampling yields sufficiently robust and clinically actionable molecular signals. Importantly, falloposcopic strategies hold promise for reducing reliance on prophylactic salpingectomy, which would mitigate associated side effects for otherwise healthy women[28–32] and preserve fertility in pre-menopausal individuals. Future research will be essential to evaluate these possibilities in a systematic and clinically rigorous manner.

**4. STIC lesion rarity and implications for retrospective detection of undiagnosed lesions**

Detection of STIC lesions in high-risk patients where the original SEE-FIM protocol did not detect any STICs is rare, as illustrated by Rabban et al.[33], who reported no identification of additional STIC lesions upon deeper FFPE tissue sections of >100 high-risk *BRCA*-mutation carriers. In a separate study in patients with pelvic carcinoma[34], an additional 25% of STIC lesions not picked up by the initial SEE-FIM protocol were identified through deeper FFPE tissue sectioning, leading to STIC identification in 23.9% of patients compared to an initial 18.0%. Once discrepancies in STIC incidence are accounted for, this further supports minimal additional detection of STIC lesions in high-risk cohorts. Assuming the highest reported percentage of high-risk patients (7%)[35] are diagnosed with a STIC using the gold-standard SEE-FIM protocol, and making the conservative assumption that the rate of additional undiagnosed STIC lesions in ovarian cancer patients applies equally to the high-risk, cancer-free cohort, only an estimated further 1.9% of high-risk patients initially negative for STIC by SEE-FIM would be diagnosed with a STIC lesion after additional deeper sectioning. This estimate is in line with a large multi-centre study by Visvanathan et al., 2018[36], which reported that although enhanced sampling increased overall detection of tubal lesions (STICs, STILs, and invasive carcinoma) from 6.3% to 11.9% in patients at high-risk of ovarian cancer (*BRCA1/2* mutation or familial risk), specific detection of STIC lesions increased from 3.5% with standard SEE-FIM to approximately 5.7% with deeper sectioning and expert re-review, a modest absolute increase of 2.2%. In the context of our high-risk cohort, this would mean of the 7 patients with either a *BRCA1* or *BRCA2* mutation who were negative for a STIC with initial SEE-FIM, an estimated 0.13-0.15 patients would have a STIC identified on deeper sectioning, reinforcing that retrospective identification of a patient harbouring an initially missed STIC in our study is highly unlikely to have occurred by chance.

**5. Future stratified cohorts**

The 82 candidate proteins identified in this study were derived from a mixed cohort comprising normal, *BRCA1/2*-mutated, and ovarian neoplasm-affected fallopian tubes. This inclusive design aimed to capture early molecular alterations across a spectrum of neoplastic risk and to evaluate whether lesion-associated signals can be detected away from the distal fimbriae, where pre-

invasive lesions arise but are delicate and difficult to access *in vivo*. As the aim of this feasibility work was to determine whether proximal tubal lavage fluid contains detectable molecular signatures associated with early tubal transformation – rather than to construct a comprehensive classifier – we focussed on proteins upregulated in both high-risk and neoplasm-adjacent groups as an initial exploratory readout of biological signal.

Future stratified cohorts will be essential to rigorously test and refine these observations. Larger, prospectively collected datasets will enable systematic evaluation of both elevated and depleted proteins, allow robust statistical testing, and clarify whether candidate markers reliably distinguish unilateral and bilateral early lesions across defined genetic risk groups. In particular, paired analysis of both tubes from individuals with unilateral disease will be invaluable for dissecting within-patient variation and refining early-detection signatures. Such studies will require sufficiently powered cohorts with adequate numbers of unilateral neoplasm cases and matched contralateral controls, recognising that these represent rare and highly informative specimens.

A key next step will involve *BRCA1/2* mutation carriers undergoing risk-reducing salpingo-oophorectomy, including individuals with and without STIC lesions. These cohorts will allow direct assessment of whether lavage-based proteomic signatures can consistently differentiate early lesions from histologically normal high-risk epithelium. Hormonal status – including menstrual cycle phase in premenopausal women – should be systematically recorded and integrated into future analyses, as endocrine regulation likely influences tubal fluid composition[37–39]. Although no clustering by menopausal status was observed in this pilot study, it was not powered to evaluate hormonal effects in detail.

Future cohorts should also incorporate chemotherapy-naïve neoplasm-adjacent fallopian tubes wherever available, as these represent ideal positive controls for defining disease-associated proteomic changes independent of treatment-related effects. While most advanced HGSOC cases in current clinical practice undergo interval debulking following neoadjuvant chemotherapy, prospectively collecting untreated tissue will be valuable for establishing baseline disease signatures.

Because the present feasibility cohort relied on *ex vivo* tissue obtained surgically, future *in vivo* sampling approaches, particularly trans-cervical, falloposcopy-based techniques will be less prone for blood contamination (see "3. Next-generation falloposcopy for early detection of pre-invasive HGSOC lesions" section above), systematic assessment of this, including quantifying its extent, variability, and impact on the luminal proteome under clinical sampling conditions, will be required.

Control cohorts will also need to include non-cancer-associated fallopian tubes from patients undergoing benign gynaecological procedures to determine whether other pelvic conditions – including non-HGSOC malignancies – alter the local proteomic milieu (see also "1. Choice of low-risk fallopian tube control samples" section above).

Adequately powered future cohorts will also enable comprehensive assessments of variation across demographic and biological factors, including age, ethnicity, menstrual cycle phase, germline mutation type, lesion laterality, and the presence or absence of occult precursor lesions. Such datasets will support more advanced analytical approaches, including dimensionality reduction, clustering, multi-omic integration, and benchmarking against external reference datasets, to refine the biological context of early tubal changes.

Future cohorts will also be needed to determine whether distal ovarian tumours generate detectable proximal tubal signals in the absence of STIC, or whether lavage-based proteomic alterations truly reflect early tubal lesions.

Finally, integration of proteomics with complementary molecular layers, such as DNA methylation profiles, extracellular vesicle cargo, and other proximal sampling readouts, may help enhance sensitivity and specificity for early detection. Prior work using for instance local sampling for endometrial cancer demonstrates the potential of integrating multiple biomarker modalities[40–43], and similar strategies may prove valuable for early tubo-ovarian cancer detection[44–48].

Together, these future prospectively designed, risk-stratified cohorts will be essential to firmly establish the reproducibility, specificity, and translational potential of lavage-based proteomic profiling as an early-detection strategy in tubo-ovarian carcinogenesis.

**References**


1. Gungor, T., Altinkaya, S. O., Ozat, M., Akbay, S. & Mollamahmutoglu, L. Unusual form of superficial spreading squamous cell carcinoma of cervix involving the endometrium, bilateral tubes and ovaries: a case report with literature review. *Arch. Gynecol. Obstet.* **283**, 323–327 (2011).
2. Shih, I. M., Wang, Y. & Wang, T. L. The Origin of Ovarian Cancer Species and Precancerous Landscape. *Am. J. Pathol.* **191**, 26–39 (2021).
3. Kurman, R. J. & Shih, I.-M. The Origin and Pathogenesis of Epithelial Ovarian Cancer: A Proposed Unifying Theory. *Am. J. Surg. Pathol.* **34**, 433–443 (2010).
4. Kurman, R. J. & Shih, I.-M. The Dualistic Model of Ovarian Carcinogenesis: Revisited, Revised, and Expanded. *Am. J. Pathol.* **186**, 733–747 (2016).
5. Doorbar, J. & Griffin, H. Refining our understanding of cervical neoplasia and its cellular origins. *Papillomavirus Res.* **7**, 176–179 (2019).
6. Woodman, C. B. J., Collins, S. I. & Young, L. S. The natural history of cervical HPV infection: unresolved issues. *Nat. Rev. Cancer* **7**, 11–22 (2007).
7. Zhang, T. *et al.* Identification of cervical cancer stem cells using single-cell transcriptomes of normal cervix, cervical premalignant lesions, and cervical cancer. *eBioMedicine* **92**, 104612 (2023).
8. Labidi-Galy, S. I. *et al.* High grade serous ovarian carcinomas originate in the fallopian tube. *Nat. Commun.* **8**, 1–10 (2017).
9. Wisztorski, M. *et al.* Fallopian tube lesions as potential precursors of early ovarian cancer: a comprehensive proteomic analysis. *Cell Death Dis.* **14**, (2023).
10. Perets, R. *et al.* Transformation of the Fallopian Tube Secretory Epithelium Leads to High-Grade Serous Ovarian Cancer in Brca;Tp53;Pten Models. *Cancer Cell* **24**, 751–765 (2013).
11. Kim, J. *et al.* High-grade serous ovarian cancer arises from fallopian tube in a mouse model. *Proc. Natl. Acad. Sci.* **109**, 3921–3926 (2012).
12. Kurman, R. J. *et al.* Papillary tubal hyperplasia: the putative precursor of ovarian atypical proliferative (borderline) serous tumors, noninvasive implants, and endosalpingiosis. *Am. J. Surg. Pathol.* **35**, 1605–1614 (2011).
13. Vang, R., Shih, I. M. & Kurman, R. J. Fallopian tube precursors of ovarian low- and high-grade serous neoplasms. *Histopathology* **62**, 44–58 (2013).
14. Laury, A. R. *et al.* Fallopian Tube Correlates of Ovarian Serous Borderline Tumors. *Am. J. Surg. Pathol.* **35**, 1759–1765 (2011).
15. Li, J. *et al.* Tubal origin of 'ovarian' low-grade serous carcinoma. *Mod. Pathol.* **24**, 1488–1499 (2011).
16. Slomovitz, B. *et al.* Low-grade serous ovarian cancer: State of the science. *Gynecol. Oncol.* **156**, 715–725 (2020).
17. Hollis, R. L. Molecular characteristics and clinical behaviour of epithelial ovarian cancers. *Cancer Lett.* **555**, 216057 (2023).
18. Allahbadia, G. N. & Merchant, R. Fallopian Tube Recanalization: Lessons Learnt and Future Challenges. *Women's Heal.* **6**, 531–549 (2010).
19. Patil, M. Assessing tubal damage. *J. Hum. Reprod. Sci.* **2**, 2–11 (2009).

20. Kerin, J., Anderson, R. & Daykhovsky, L. Falloposcopy: microendoscopy of the human fallopian tube from the uterotubal junction to the fimbria using a transcervico-uterine approach. *Fertil Steril* **54**, 390 (1990).
21. Rimbach, S., Wallwiener, D. & Bastert, G. Falloposcopy: its place in the state-of-the-art spectrum of tubal investigation methods. *Fertil. Steril. A Curr. Overv. Proc. 15th World Cong. Fertil. Steril.* **13**, 97–103 (1995).
22. Wong, A. Y. K. & Walker, S. M. Falloposcopy-a prerequisite to the proper assessment of tubal infertility. *Hong Kong Med. J.* **5**, 76–81 (1999).
23. Rocha, A. D. *et al.* First Clinical Feasibility and Safety Study of a Novel Multimodality Fallopian Tube Imaging Endoscope. *Lasers Surg. Med.* 163–170 (2025) doi:10.1002/lsm.23877.
24. Cordova, R. *et al.* Sub-millimeter endoscope demonstrates feasibility of in vivo reflectance imaging, fluorescence imaging, and cell collection in the fallopian tubes. *J. Biomed. Opt.* **26**, (2021).
25. Galvez, D. *et al.* Cell-acquiring fallopian endoscope for detection of ovarian cancer via reflectance imaging, fluorescence imaging, and cell collection. in *Endoscopic Microscopy XVIII* (eds. Suter, M. J., Tearney, G. J. & Wang, T. D.) 11 (SPIE, 2023). doi:10.1117/12.2650875.
26. Schmidt, C. K., Medina-Sánchez, M., Edmondson, R. J. & Schmidt, O. G. Engineering microrobots for targeted cancer therapies from a medical perspective. *Nat. Commun.* **11**, 5618 (2020).
27. Xu, H. *et al.* Human spermbots for patient-representative 3D ovarian cancer cell treatment. *Nanoscale* **12**, 20467–20481 (2020).
28. Kershaw, V., Hickey, I., Wyld, L. & Jha, S. The impact of risk reducing bilateral salpingo-oophorectomy on sexual function in BRCA1/2 mutation carriers and women with Lynch syndrome: A systematic review and meta-analysis. *Eur. J. Obstet. Gynecol. Reprod. Biol.* **265**, 7–17 (2021).
29. Parker, W. H. *et al.* Long-Term Mortality Associated With Oophorectomy Compared With Ovarian Conservation in the Nurses' Health Study. *Obstet. Gynecol.* **121**, 709–716 (2013).
30. Fakkert, I. E. *et al.* Bone mineral density and fractures after risk-reducing salpingo-oophorectomy in women at increased risk for breast and ovarian cancer. *Eur. J. Cancer* **51**, 400–408 (2015).
31. Rivera, C. M. *et al.* Increased cardiovascular mortality after early bilateral oophorectomy. *Menopause* **16**, 15–23 (2009).
32. Gaba, F. & Manchanda, R. Systematic review of acceptability, cardiovascular, neurological, bone health and HRT outcomes following risk reducing surgery in BRCA carriers. *Best Pract. Res. Clin. Obstet. Gynaecol.* **65**, 46–65 (2020).
33. Rabban, J. T. *et al.* Multistep level sections to detect occult fallopian tube carcinoma in risk-reducing salpingo-oophorectomies from women with BRCA mutations: Implications for defining an optimal specimen dissection protocol. *Am. J. Surg. Pathol.* **33**, 1878–1885 (2009).
34. Mahe, E. *et al.* Do deeper sections increase the frequency of detection of serous tubal intraepithelial carcinoma (stic) in the sectioning and extensively examining the fimbriated end (see-fim) protocol? *Int. J. Gynecol. Pathol.* **32**, 353–357 (2013).
35. Luvero, D. *et al.* Serous Tubal Intraepithelial Carcinoma (STIC): A Review of the Literature on the Incidence at the Time of Prophylactic Surgery. *Diagnostics* **14**, 1–11 (2024).
36. Visvanathan, K. *et al.* Fallopian tube lesions in women at high risk for ovarian cancer: A multicenter study. *Cancer Prev. Res.* **11**, 697–705 (2018).
37. Beddows, I. *et al.* Impact of BRCA mutations , age , surgical indication , and hormone status on the molecular phenotype of the human Fallopian tube. *Nat. Commun.* (2025) doi:10.1038/s41467-025-58145-2.
38. Weigert, M. *et al.* A cell atlas of the human fallopian tube throughout the menstrual cycle and menopause. *Nat. Commun.* **16**, 372 (2025).
39. Sipes, J. *et al.* Spatial transcriptomic profiling of the human fallopian tube epithelium reveals region-specific gene expression patterns. *Commun. Biol.* **8**, 520 (2025).
40. Njoku, K. *et al.* Detection of endometrial cancer in cervico-vaginal fluid and blood plasma: leveraging proteomics and machine learning for biomarker discovery. *eBioMedicine* **102**, 105064 (2024).
41. Njoku, K. *et al.* Quantitative SWATH-based proteomic profiling of urine for the identification of endometrial cancer biomarkers in symptomatic women. *Br. J. Cancer* **128**, 1723–1732 (2023).
42. Herzog, C. *et al.* A Simple Cervicovaginal Epigenetic Test for Screening and Rapid Triage of Women With Suspected Endometrial Cancer: Validation in Several Cohort and Case/Control

Sets. *J. Clin. Oncol.* **40**, 3828–3838 (2022).
43. Bakkum-Gamez, J. N. *et al.* Detection of endometrial cancer via molecular analysis of DNA collected with vaginal tampons. *Gynecol. Oncol.* **137**, 14–22 (2015).
44. Galey, M. M. *et al.* Detection of Ovarian Cancer Using Samples Sourced from the Vaginal Microenvironment. *J. Proteome Res.* **19**, 503–510 (2020).
45. Ghezelayagh, T. S. *et al.* Uterine Lavage Identifies Cancer Mutations and Increased TP53 Somatic Mutation Burden in Individuals with Ovarian Cancer. *Cancer Res. Commun.* **2**, 1282–1292 (2022).
46. Wever, B. M. M. *et al.* Molecular analysis for ovarian cancer detection in patient-friendly samples. *Commun. Med.* **4**, (2024).
47. Medina, J. E. *et al.* Early Detection of Ovarian Cancer Using Cell-Free DNA Fragmentomes and Protein Biomarkers. *Cancer Discov.* **15**, 105–118 (2025).
48. Barr, C. E., Njoku, K., Owens, G. L. & Crosbie, E. J. Urine CA125 and HE4 for the Detection of Ovarian Cancer in Symptomatic Women. *Cancers (Basel).* **15**, 1–13 (2023).
