# Supplementary material for: Fallopian tube lavage sampling towards early detection of pre‐invasive ovarian cancer
Source: Clin Transl Med. 2026 Jan 2;16(1):e70557. doi: 10.1002/ctm2.70557 (PMC12759042; doi:10.1002/ctm2.70557)
Supplement: Supplementary file 2 — Supporting Information. [file CTM2-16-e70557-s004.pdf]

Supplementary Figure S1

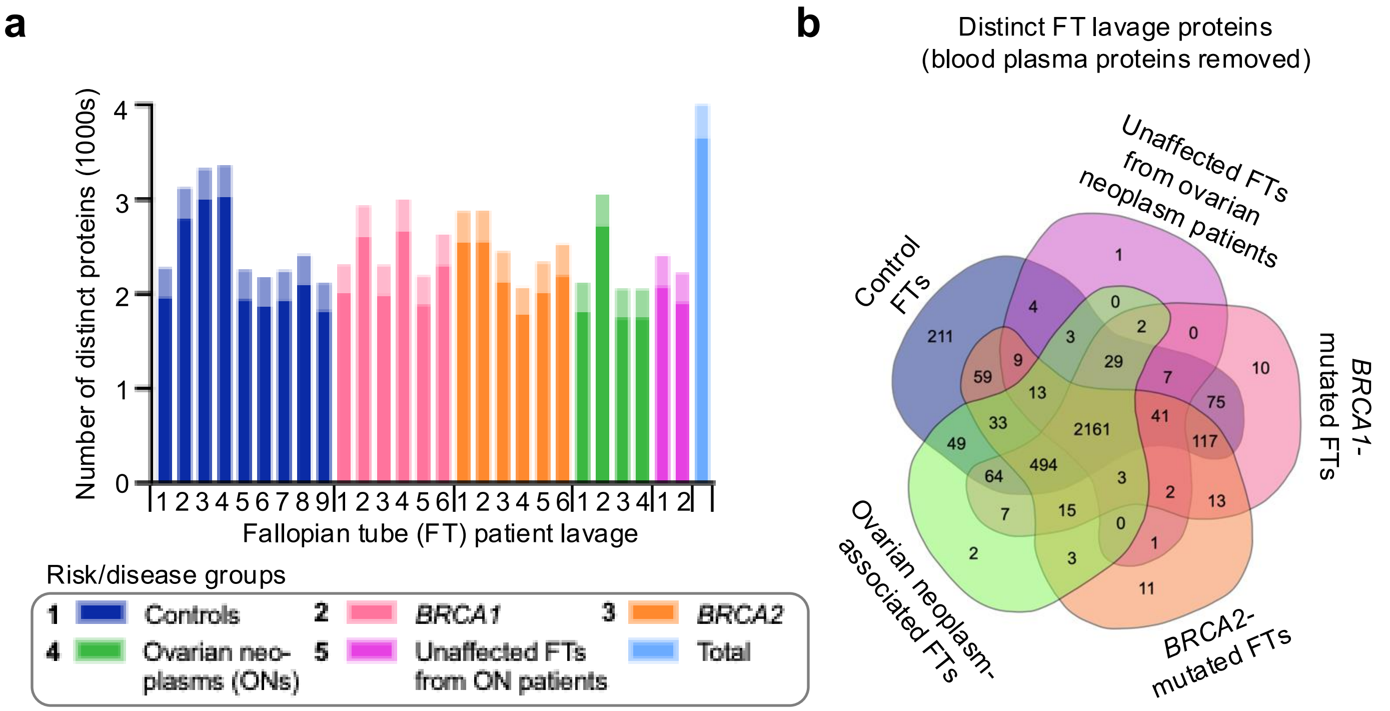

Supplementary Figure S1: Establishing fallopian tube lavage samples for proteomic analysis. (**a**) Comparison of levels and numbers of distinct fallopian tube (FT) lavage proteins identified by LC-MS between samples belonging to the different risk/disease groups as indicated. Pale segments at the top of the bars highlight the number of top blood proteins detected in each sample which were subsequently eliminated before downstream analysis. (**b**) Venn diagram showing the number of distinct proteins detected per patient group after removal of abundant blood proteins. Abbreviations: FT: fallopian tube; LC-MS: liquid chromatography-mass spectrometry.

Supplementary Figure S2

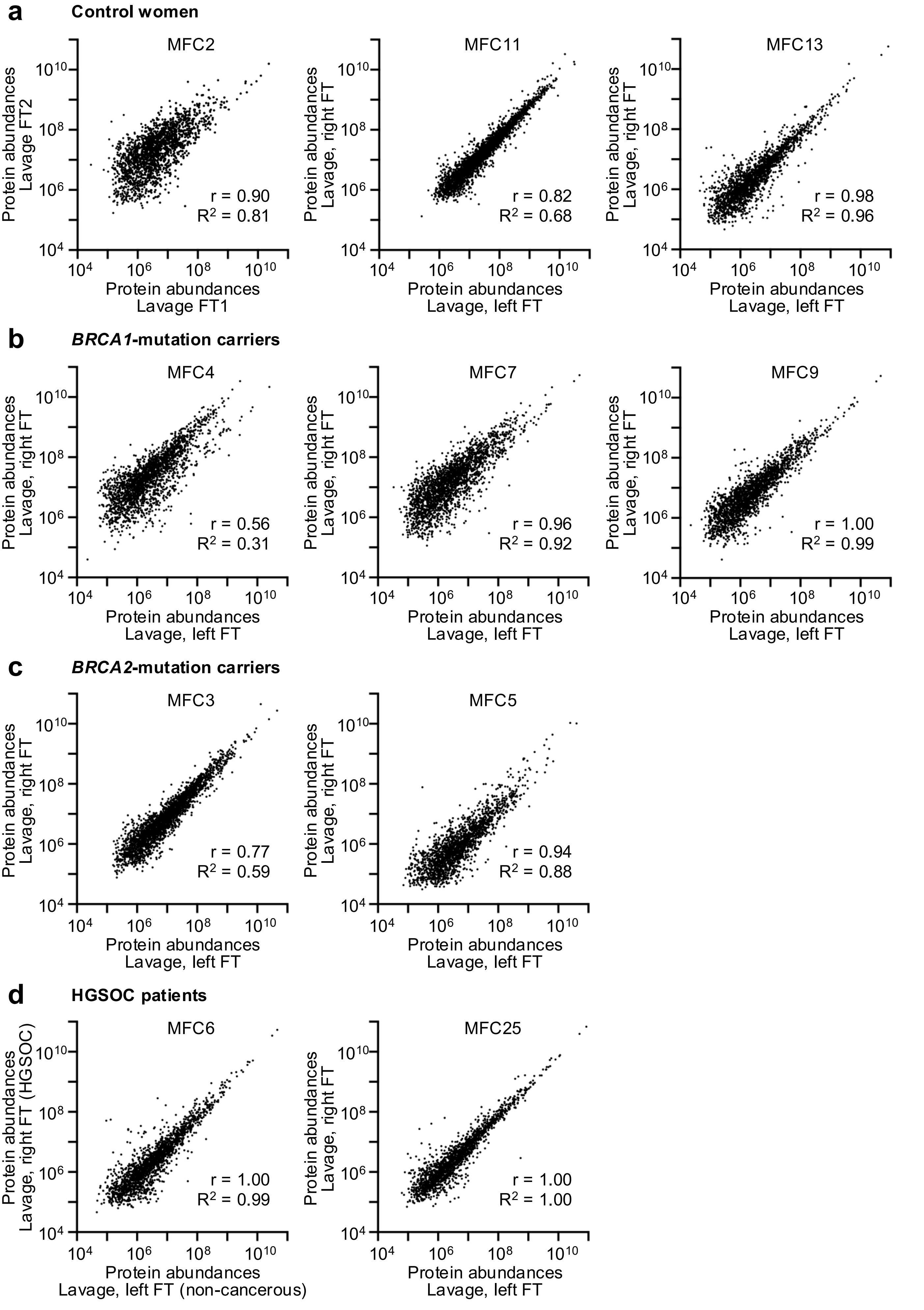

Supplementary Figure S2: Correlation analyses for patients for whom a sample was collected from both fallopian tubes. (**a**) Correlation analyses for patients for whom a sample was collected from both fallopian tubes (FTs)**.** Correlations of detected proteins for FT lavage samples obtained from both FTs of control patients. (**b**) Same as for (a) but for *BRCA1*-mutation carriers. (**c**) Same as for (a) but for *BRCA2*-mutation carriers. (**d**) Same as for (a) but for patients diagnosed with an ovarian neoplasm. The Pearson correlation coefficients (r) and R^2 values were calculated for each pair of samples using GraphPad Prism 10.0.2. Correlation analysis of MCF6 also forms part of the main Figures (**Fig. 2b**), as indicated there. Clinical information for all patients can be found in **Figure 1a**. Abbreviations: FT: fallopian tube.

Supplementary Figure S3

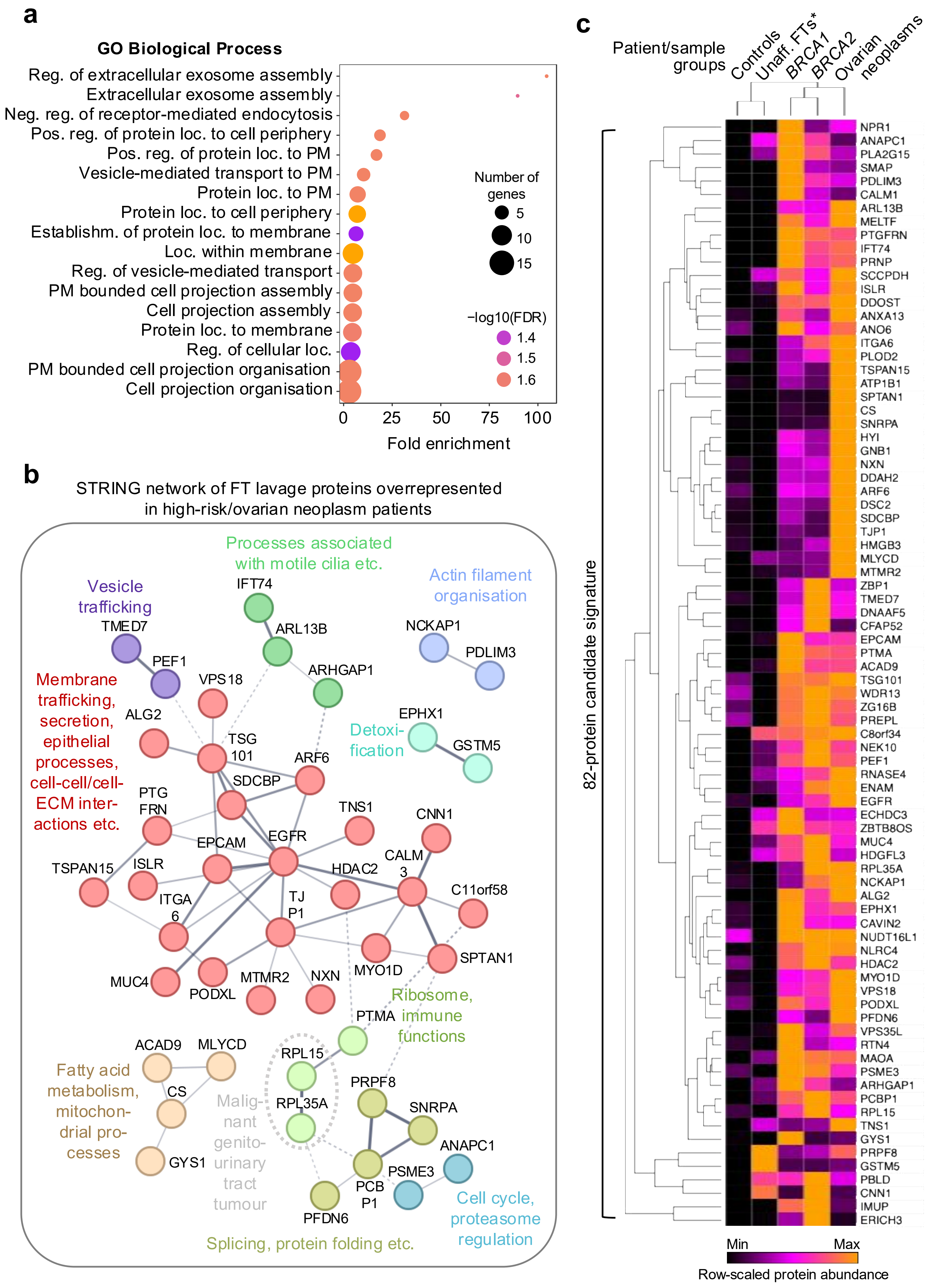

Supplementary Figure S3: Enrichment, network and hierarchical clustering analysis of 82-protein signature. (**a**) Gene ontology (GO) analysis (Biological Process aspect) of 82 proteins overrepresented in high-risk and ovarian neoplasm patients compared to controls, highlighting membrane proteins as a highly enriched fraction amongst the candidate proteins. (**b**) STRING analysis of 82 proteins overrepresented in high-risk and ovarian neoplasm patients compared to controls, highlighting a significantly enriched interaction network centred around EGFR, a key oncogene, and including proteins, such as EPCAM, MUC4, IFT74, CALM3 and EGFR known to be linked to the fallopian tube epithelium[1]. (**c**) Hierarchical clustering of 82 overrepresented proteins, illustrating different profiles between the two control groups – samples from control patients (MFC2, 11, 12, 13, 14 and 17), and unaffected fallopian tubes (FTs, *) from ovarian neoplasm patients (MFC6, 21) – and the samples from patients either at high-risk (MFC4, 7 and 9 for *BRCA1*; MFC3, 5, 8 and 10 for *BRCA2*) of ovarian cancer or associated with an ovarian neoplasm (MFC6, 23 and 25). For patient information see Figure 1a. Note the overarching trends across patient categories, showing that the control samples cluster with lavages from unaffected fallopian tubes of patients with unilateral ovarian tumours, samples that were not used in defining the 82-protein candidate signature. Colour range indicates abundance of proteins as detected by mass spectrometry, based on a relative colouring scheme, using the minimum and maximum values in each row. Schematic generated using Morpheus (https://software.broadinstitute.org/morpheus). Abbreviations: EGFR: epidermal growth factor receptor; Establishm.: establishment; FDR: false discovery rate; FT: fallopian tube; GO: gene ontology; loc.: localisation; Neg.: negative; PM: plasma membrane; Pos.: positive; Reg.: regulation; Unaff.: unaffected.

Supplementary Figure reference

1. Uhlén et al. Tissue-based map of the human proteome. Science 347, 1260419 (2015). DOI:10.1126/science.1260419; proteinatlas.org, accessed 25/11/25).
