# Supplementary material for: Fallopian tube lavage sampling towards early detection of pre‐invasive ovarian cancer
Source: Clin Transl Med. 2026 Jan 2;16(1):e70557. doi: 10.1002/ctm2.70557 (PMC12759042; doi:10.1002/ctm2.70557)
Supplement: Supplementary file 3 — Supporting Information. [file CTM2-16-e70557-s002.pdf]

SUPPLEMENTARY METHODS

Human sample collection

Fallopian tube (FT) lavages and formalin-fixed paraffin-embedded (FFPE) FT tissues were obtained via the Manchester University NHS Foundation Trust (MFT) Biobank under Human Tissue Authority (HTA)-approved ethics applications (REC14/NW/1260 and REC19/NW/0644). All patients provided their written informed consent and were prospectively included in the study. FT lavages were collected in the operating room from women aged 33–78 (no significant patient age differences between the five groups; p=0.115, ANOVA) undergoing salpingo-oophorectomy or hysterectomy. After surgical removal, the FTs were cut ~2 cm from the utero-tubal junction, and a syringe containing sterile PBS was inserted into the fimbrial end with the syringe tip positioned just beyond the fimbrial ostium into the tubal lumen. 5 mL of sterile PBS were introduced gently, without applying pressure, instrumentation or traction, effectively flushing the infundibulum, ampulla and isthmus regions whilst avoiding the fimbrae, and the fluid collected. Samples were immediately put onto ice and stored at -80 °C within 10 min.

Sample preparation and mass spectrometry (MS) workflow

Lavages were concentrated by spinning through Vivaspin turbo 4 (3,000 MWCO) ultrafiltration units (Sartorius; Göttingen, Germany) for 20 min at 4 °C, 4,000 g, followed by plasma protein depletion using Thermo Scientific High-Select™ Top14 Abundant Protein Depletion Resin (Massachusetts, US). Samples were processed for MS using established University of Manchester BioMS Core Facility protocols:

- DOI: 10.17504/protocols.io.261genmkdg47/v1[1]

In short, samples were reduced with dithiothreitol, then alkylated with iodoacetamide and clarified by centrifuging at 14,000 g for 10 min. Trypsin digestion was performed in-column using S-Trap columns (ProtiFi; New York, US), followed by desalting with Oligo R3 resin beads (Thermo Fisher; Massachusetts, US).

Samples were analysed on an UltiMate® 3000 Rapid Separation LC system (RSLC, Thermo Fisher; Massachusetts, US) connected to an Exploris 480 mass spectrometer (Thermo Fisher; Massachusetts, US) via a nanospray Flex Ion source (Thermo Fisher; Massachusetts, US). Peptides were separated on a Waters nanoEase M/Z Peptide CSH C18 Column (130 Å, 1.7 µm, 75 µm x 250 mm; Massachusetts, US) using a 75 min multistage gradient. MS data were acquired in a data-dependent manner in positive mode (scan range: 300–1750 Th) with a charge state of +2 or +3, an intensity over 5,000, and dynamic exclusion set at 15 s. The MS resolution was set at 120,000 with a normalised automatic gain control (AGC) target of 300% and a maximum fill time set at 25 ms. The MS2 resolution was set to 15,000, with a normalised collision energy of 30%, a normalised AGC target of 300%, first mass of 110 Th and a maximum fill time of 25 ms.

Fallopian tube lavage mass spectrometry proteomics data are accessible through the PRIDE repository (accession number: PXD072299).

Liquid chromatography (LC) separation (loop out). Separation was performed on a Thermo RSLC system (Massachusetts, US) consisting of a NCP3200RS nanopump, WPS3000TPS autosampler and TCC3000RS column oven configured with buffer A as 0.1% formic acid in water and buffer B as 0.1% formic acid in acetonitrile. An injection volume of 2 µL was used to load into the end of a 5 µL loop and reverse flushed on to the analytical column (Waters nanoEase M/Z Peptide CSH C18 Column, 130 Å, 1.7 µm, 75 µm x 250 mm; Massachusetts, US), kept at 35 °C at a flow rate of 300 nL/min for 8 min with an initial pulse of 500 nL/min for 0.3 min to rapidly re-pressurise the column. The injection valve was set to load before a separation consisting of a multistage gradient of 1% B to 6% B over 2 min, 6% B to 18% B over 44 min, 18% B to 29% B over 7 min and 29% B to 65% B over 1 min before washing for 4 min at 65% B and dropping down to 2% B in 1 min. The complete method time was 75 min.

Mass spectrometry source. The analytical column was connected to a Thermo Exploris 480 mass spectrometry system via a Thermo nanospray Flex Ion source via a 20 µm ID fused silica capillary (Massachusetts, US). The capillary was connected to a fused silica spray tip with an outer diameter of 360 µm, an inner diameter of 20 µm, a tip orifice of 10 µm and a length of 63.5 mm (New Objective Silica Tip FS360-20-10-N-20-6.35CT; Massachusetts, US) via a butt-to-butt connection in a steel union using a custom-made gold frit (Agar Scientific AGG2440A; Rotherham, UK) to provide the electrical connection. The nanospray voltage was set at 1900 V and the ion transfer tube temperature set to 275 °C.

Mass spectrometry settings. Data were acquired in a data-dependent manner using a fixed cycle time of 1.5 s, an expected peak width of 15 s and a default charge state of 2. Quantification was performed using label-free precursor-intensity-based quantification in Proteome Discoverer (PD) software (Thermo Fisher; Massachusetts, US; version 2.5.0.400; RRID:SCR_014477) (see Label-free quantification section). Full MS data were acquired in positive mode over a scan range of 300 to 1750 Th, with a resolution of 120,000, a normalised AGC target of 300% and a max fill time of 25 ms for a single microscan. Fragmentation data were obtained from signals with a charge state of +2 or +3 and an intensity over 5,000, and they were dynamically excluded from further analysis for a period of 15 s after a single acquisition within a 10 ppm window. Fragmentation spectra were acquired with a resolution of 15,000 with a normalised collision energy of 30%, a normalised AGC target of 300%, first mass of 110 Th and a max fill time of 25 ms for a single microscan. All data were collected in profile mode.

MS data analysis. Raw spectra were processed using PD, with a target false discovery rate (FDR) cutoff of 0.01. Blood contaminants were excluded based on the top 400 blood plasma proteins[2]. Label-free quantification was enabled in PD to calculate precursor and protein abundances as described below. The processing workflow was set to search 2,370,091 spectra against the protein database *Homo sapiens* (sp_canonical TaxID=9606) (v2021-03-31) (20,325 sequences) using the Sequest HT software (version provided with PD). The protein identification search engine parameters used were:

- Trypsin (cleaving at lysines and arginines except where the presence of a C-terminal proline obstructed cleavage)
- Precursor tolerance of 10 ppm and fragmentation tolerance of 0.02 Da
- Fixed modifications of carbamidomethyl (+57.021 Da) to cysteine and variable modifications of oxidation (+15.995 Da) to methionine

A false discovery rate (FDR) was calculated for both the protein level and the peptide level by Proteome Discoverer. Proteins were labelled with high confidence where the FDR was less than 0.01; medium confidence where the FDR was between 0.01 and 0.05; and low confidence where the FDR was greater than 0.05.

Label-free quantification and calculation of protein abundance. All protein quantification was performed using the label-free quantification workflow implemented in PD, which is fully compatible with data-dependent acquisition (DDA) on the Exploris 480 system used in this study. After peptide identification, all samples were aligned along the retention-time axis to enable transfer of quantitative information between runs, allowing PD to assign precursor-ion intensities to features that did not receive an MS/MS event in a given sample, based on confidently identified counterparts in other samples.

Precursor-ion quantification was based on peak intensity (peak height) rather than area-under-the-curve. Peptide abundances were normalised using PD's "total peptide amount" mode, applying a sample-wise normalisation factor calculated across all detected peptides. Pairwise protein ratios were calculated excluding modified peptides. Protein-level abundance values were generated by summing peptide-level abundances as implemented in PD (summed intensity across peptides, no missing-value imputation was applied). A background-based t-test was used

by PD to calculate p-values when requested by the software, although for the purposes of this feasibility study, no statistical significance thresholds were applied. A maximum fold-change cap of 100 was used. Minimum peptide length was set to 6 amino acids, with a minimum of one peptide required for protein reporting. Statistical metrics per protein match, such as FDR q values, sum posterior error probability (PEP) scores, % coverage, number of peptides, number of peptide spectrum matches (PSMs) and number of unique peptides, are provided in Supplementary Table S1 to facilitate data interpretation.

This commercially maintained workflow is Thermo-validated and widely used for quantitative analysis of label-free DDA proteomics data and was selected for compatibility with both the instrumentation and institutional data-analysis pipelines.

Throughout the manuscript, terms such as 'enriched' or 'overexpressed' are used to describe relative protein abundance in individual samples. These are descriptive observations and do not imply statistical significance, particularly in single-patient comparisons."

Data visualisation and interpretation. Morpheus (RRID:SCR_014975) was used for generating heatmaps and hierarchical clustering: https://software.broadinstitute.org/morpheus. A value of '0' was assigned to non-detected proteins. To calculate minimum protein abundance ratios, values of '1' were used for non-detected proteins. Venn diagrams were generated using:

- http://bioinformatics.psb.ugent.be/webtools/Venn/.

Functional protein association network and enrichment analyses were performed using STRING v11.5 (RRID:SCR_005223)[3], with required confidence score and FDR stringency set to medium, ShinyGO v0.80 (RRID:SCR_019213)[4], with settings set to default, and the ToppGene Suite (RRID:SCR_005726)[5], using default settings and DisGeNET (RRID:SCR_006178)[6] and the Alliance of Genome Resources (RRID:SCR_015850)[7] as integrated sources.

Immunohistochemistry (IHC) and immunofluorescence (IF)
For all applications, FFPE sections (4 µm) were mounted on charged slides. For IHC, Ki67 or p53, staining was run on a Ventana Discovery Ultra (Roche; Basel, Switzerland; RRID:SCR_021254), using the ultraView Universal DAB Detection kit for 32 min at 37 °C or the DISCOVERY OmniMap anti-Rb HRP and Chromomap DAB kits for 16 min, respectively. Slides were dewaxed onboard, and antigen retrieval was performed using CC1 solution (pH 8.74) for 36 min. Slides were counterstained with Haematoxylin II for 12 min, and bluing reagent for 8 min. Slides were then rehydrated, cleared and coverslipped. Haematoxylin and eosin (H&E) staining was run on a Leica Autostainer XL Automated Slide Stainer (Wetzlar, Germany; RRID:SCR_020212). Whole-slide images were captured with an Olympus VS200 Slide Scanner (Tokyo, Japan; RRID:SCR_024783).

IF staining was carried out on a Leica BOND RX Stainer (Wetzlar, Germany; RRID:SCR_025548), with automated dewaxing and heat-induced epitope retrieval of slides using Epitope Retrieval Solution 1 (ER1) for 20 min at 100 °C. ITGA6, EPCAM and EGFR staining was carried out with the Research Detection System 2. All primary and secondary antibodies were applied for 30 min. Signal amplification was performed for ITGA6 by staining with TSA650 for 30 min. All slides were counterstained with 0.33 µg/mL DAPI for 15 min and coverslipped using ProLong Gold Antifade mountant (Thermo Fisher). Whole-slide images were captured with an Olympus VS120 microscope (Tokyo, Japan; (RRID:SCR_018411).

Fluorescence staining was quantified using ImageJ (RRID:SCR_003070). For median fluorescence comparisons, fluorescent intensities were measured along lines drawn across normal FT epithelium (FTE) and STICs. Fluorescence intensities were background corrected and normalised to normal adjacent FTE, and median fluorescence calculated per STIC and its paired normal FTE. One-tailed Wilcoxon matched-pairs signed-rank tests were used to evaluate differences between normal FTE and STIC lesions for ITGA6 and EPCAM, as well as between apical and basal regions of both normal FTE and STIC lesions for EGFR. For EGFR comparisons

across the apical-to-basal axis, lines were drawn from just outside the apical surface of the cell to just below the basal region and fluorescence intensity quantified for each pixel along the line. Five regions were quantified for both normal FTE and STIC. Average fluorescence intensity and standard deviation were then calculated for normal FTE and STIC. Statistical analyses and graphs were generated using GraphPad Prism 10.0.2 (RRID:SCR_002798).

Antibodies used

For immunohistochemistry:

- Anti-p53 (Agilent, M7001, RRID: AB_2206626, 1:100)
- Anti-Ki67 (Abcam, ab16667, RRID: AB_302459, 1:300)

For immunofluorescence (primary antibodies):

- Anti-ITGA6 (Atlas Antibodies, HPA012696, RRID: AB_1851822, 1:400)
- Anti-EGFR (R&D Systems, AF231, RRID: AB_3552, 1:100)
- Anti-RRAS2 (Atlas Antibodies, HPA050942, RRID: AB_2681285, 1:50)
- Anti-EPCAM (Santa Cruz Biotechnology, sc-59906, RRID: AB_783252, 1:400)

For immunofluorescence (secondary antibodies):

- Anti-mouse Alex Fluor 594 (Invitrogen, A-11032, RRID: AB_2534091, 1:400)
- Anti-goat Alexa Fluor 647 (Invitrogen, A-21447, RRID: AB_141844, 1:400)
- Anti-rabbit Alexa Fluor 750 (Invitrogen, A-21039, RRID: AB_2535710, 1:400)

Whole-slide scanning

Immunohistochemistry (IHC) was performed using the Olympus VS200 MTL (Olympus Tokyo, Japan), in conjunction with an Olympus UPLXAPO 20 x objective (NA 0.6): 0.274 μm/pixel. Focus points were chosen to permit a well-defined image across the whole tissue section. The images were captured on multiple axial planes, and then combined using a depth of focus algorithm to maintain focus and render a three-dimensional image into two dimensions. Images were captured using an Orca-Flash4 camera (Hamamatsu Photonics, Germany) where each field of view was captured at 2048 x 2048 pixels, all under control by the Olympus ASW software.

For IF slides, an Olympus VS120-L100-W-12 (Olympus Corporation, Tokyo, Japan) was utilised under fluorescence illumination using a Lumencor SOLA LED light source and a fast filter wheel equipped with an Olympus penta AHF-SPX-QSEM filter set (DAPI, FITC, TRITC, Cy5 and Cy7). An Olympus UPLSAPO 20 x objective (NA 0.75) was used to capture the data (digitisation via a sCMOS ORCA FLASH v4.0V3 camera, 2048 x 2048 pixels with 6.5 x 6.5 μm cell size (Hamamatsu, Shizuoka Pref, Japan) with Peltier cooling down to -10 °C) and equipment/acquisition controlled using the Olympus VS-ASW software.

References

1. CRF, B. & ronan o'cualain. Researcher led sample preparation for LC-MS using the BioMS research core facility. *protocols.io* (2022).
2. Geyer, P. E. *et al.* Plasma Proteome Profiling to Assess Human Health and Disease. *Cell Syst* 2, 185–195 (2016).
3. Szklarczyk, D. *et al.* STRING v11: Protein-protein association networks with increased coverage, supporting functional discovery in genome-wide experimental datasets. *Nucleic Acids Res* 47, D607–D613 (2019).
4. Ge, S. X., Jung, D., Jung, D. & Yao, R. ShinyGO: A graphical gene-set enrichment tool for animals and plants. *Bioinformatics* 36, 2628–2629 (2020).
5. Chen, J., Bardes, E. E., Aronow, B. J. & Jegga, A. G. ToppGene Suite for gene list enrichment analysis and candidate gene prioritization. *Nucleic Acids Res* 37, 305–311 (2009).
6. Piñero, J. *et al.* The DisGeNET knowledge platform for disease genomics: 2019 update. *Nucleic Acids Res* 48, D845–D855 (2020).
7. Aleksander, S. A. *et al.* Updates to the Alliance of Genome Resources central infrastructure. *Genetics* 227, 1–18 (2024).
