# Supplementary material for: Fallopian tube lavage sampling towards early detection of pre‐invasive ovarian cancer
Source: Clin Transl Med. 2026 Jan 2;16(1):e70557. doi: 10.1002/ctm2.70557 (PMC12759042; doi:10.1002/ctm2.70557)
Supplement: Supplementary file 5 — Supporting Information. [file CTM2-16-e70557-s003.pdf]

Gene symbol	Protein Description	Reported roles in cancer (roles reported in ovarian cancer highlighted in bold)	Fold-change (BRCA1 mut)	Fold-change (BRCA2 mut)	Fold-change (ovarian neoplasm)
PTGFRN	Prostaglandin F2 receptor negative regulator	**More frequently observed in ovarian cancer plasma samples than in controls** (1); **Interacts with potential ovarian cancer target TSPAN15** (also identified in our samples) (2); Upregulated in glioblastoma, correlated with poor prognosis (3)	231.0	194.0	175.1
IFT74	Intraflagellar transport protein 74 homologue	Involved in cilia and primary cilium function (4); **Aberrant primary cilium function linked to epithelial ovarian cancer** (5) and other cancers, with a potential role in drug resistance (6)	234.1	170.5	195.4
ARL13B	ADP-ribosylation factor-like protein 13B	Overexpressed in breast and gastric cancer and promotes proliferation, migration and invasion (7, 8); upregulated in breast cancer cell lines and patient tissue samples (9)	166.2	139.2	294.6
PRNP	Major prion protein	Identified as a potential therapeutic target in cancer (10, 11); Associated with poor prognosis and resistance to treatment in breast, colon and gastric cancer (12); Evidence for involvement in cancer cell proliferation, migration and invasion, therapeutic resistance and cancer stem cell properties (13)	202.4	138.6	163.9
MELTF	Melanotransferrin	Prognostic marker (unfavourable) in lung, pancreatic, renal (proteinatlas.org, 14) and gastric cancer (15); potential tissue and serum biomarker for gastric cancer (15) and serum biomarker for colorectal cancer (16)	220.7	136.8	242.5
SCCPDH	Saccharopine dehydrogenase-like oxidoreductase	Gene expression in primary melanomas associated with short survival (17)	176.3	112.1	217.1
ITGA6	Integrin alpha-6	**Linked to increased proliferation and metastasis of ovarian cancer cells** (18); **potential antigen for targeted therapy in ovarian cancer** (19); **upregulation confers platinum therapy resistance, secretion contributes to pre-metastatic niche enabling epithelial ovarian cancer dissemination** (20)	109.3	218.5	272.3
ZBP1	Z-DNA-binding protein 1	**Overexpressed in ovarian cancer cell lines and potentially predictive of ovarian cancer prognosis** (21); **Mediates necroptosis in ovarian cancer** (22) **and part of ovarian cancer programmed cell death signature** (21); Enhancer regions more active in BRCA1-mutated cells compared to BRCA1-wildtype (23); Deletion inhibits tumour metastasis in a breast cancer model (24)	111.3	247.3	105.0
PDLIM3	PDZ and LIM domain protein 3	**Strong correlation between RNA expression and overall and progression-free survival in ovarian cancer patients** (25); **Upregulated in metastatic versus primary ovarian serous papillary carcinoma** (26); Upregulation associated with poor prognosis, immune cell infiltration, and activation of two key signal pathways in gastric cancer (27); Prognostic marker (unfavourable) in renal, thyroid and liver cancer (proteinatlas.org, 14)	222.5	143.7	100.0
ALG2	Alpha-1,3/1,6-mannosyltransferase ALG2	Identified role in cancer cell proliferation, migration and tumorigenicity (28); Dysregulated in tumours, plays an important role in maintaining cancer cell viability, upregulated in lung cancer (29)	135.6	94.0	140.0
NPR1	Atrial natriuretic peptide receptor 1	**Upregulated in ovarian cancer-associated omentum tissue** (30); **Associated with shorter relapse-free survival in ovarian cancer** (31); Prognostic (unfavourable) marker in endometrial cancer (proteinatlas.org, 14)	336.7	87.6	175.7
SMAP	Small acidic protein	Unfavourable prognostic marker in liver cancer (proteinatlas.org, 14)	285.0	106.2	81.3
DNAAF5	Dynein assembly factor 5, axonemal	Prognostic marker in liver cancer (unfavourable) and head and neck cancer (unfavourable) (proteinatlas.org, 14)	75.1	145.1	81.0
HYI	Putative hydroxypyruvate isomerase	Prognostic marker in urothelial cancer (favourable), renal cancer (unfavourable), colorectal cancer (unfavourable), liver cancer (unfavourable) and breast cancer (favourable) (proteinatlas.org, 14)	122.1	71.8	219.9
TSPAN15	Tetraspanin-15	**Identified as a biomarker for ovarian cancer metastasis** (32); **Identified as a potential targetable cell-surface marker in ovarian cancer** (33); Promotes tumour cell proliferation (34); Identified as a stemness-related marker in hepatocellular carcinoma (35)	122.2	62.8	310.5
IMUP	Immortalization up-regulated protein	Overexpression associated with tumorigenicity (36); Promotes pancreatic cancer progression (37); Expression is upregulated in pancreatic ductal adenocarcinoma and prognostic of survival (38)	248.0	296.2	55.8
ECHDC3	Enoyl-CoA Hydratase Domain Containing 3	Upregulated in CD34+ progenitors of chemo-resistant acute myeloid leukaemia and identified as a poor prognostic biomarker (39)	124.3	53.5	56.9
ANAPC1	Anaphase Promoting Complex Subunit 1	Prognostic marker (unfavourable) in endometrial cancer (proteinatlas.org, 14)	251.1	170.3	48.3
PRPF8	Pre-mRNA processing factor 8	**Increased expression and associated with proliferation and inhibition of apoptosis in ovarian cancer cells** (40); Increased in breast cancer tissue, high expression associated with poor prognosis (41)	10.0	13.6	29.0
ISLR	Immunoglobulin superfamily containing leucine-rich repeat	Prognostic marker (unfavourable) in renal and pancreatic cancer (proteinatlas.org, 14)	10.2	5.8	9.8
PBLD	Phenazine biosynthesis-like protein domain containing	Favourable prognostic marker in renal (favourable), liver (favourable), pancreatic (favourable) and prostate cancer (unfavourable) (proteinatlas.org, 14); Reduced expression in breast cancer, associated with favourable prognosis (42)	7.8	11.7	5.8
PLA2G15	Phospholipase A2 group XV	Prognostic marker in renal (unfavourable) and liver cancer (favourable) (proteinatlas.org, 14)	13.6	10.6	5.7
DDOST	Dolichyl-diphosphooligosaccharide--protein glycosyltransferase non-catalytic subunit	High expression correlates with poor prognosis of tumour patients, expression closely related to immunosuppressive tumour microenvironment (43); Prognostic marker in renal (unfavourable), liver (unfavourable), head and neck cancer (favourable) and endometrial cancer (unfavourable) (proteinatlas.org, 14)	5.6	5.3	6.5

C8orf34	Chromosome 8 open reading frame 34	Prognostic marker (favourable) in lung cancer (proteinatlas.org, 14)	5.2	5.8	6.1
PTMA	Prothymosin alpha	**Role in ovarian cancer cell adhesion, migration, and proliferation** (44); **Enriched in the urine of ovarian cancer patients** (45); **Part of a classifier in urine developed to distinguish benign and malignant ovarian tumours** (46)	5.5	4.7	4.4
GNB1	G protein subunit beta 1	**Closely related to the CXC receptor family which has been identified as a potential biomarker for ovarian cancer prognosis** (47); Upregulation of GNB1 may be a potential diagnostic biomarker for CRC patients (48)	6.3	4.4	11.7
EPHX1	Epoxide Hydrolase 1	Prognostic (favourable) in renal cancer (proteinatlas.org, 14); Methylation of promotor altered in polycystic ovary syndrome (PCOS) and involved in estradiol regulation (49)	6.4	4.2	4.9
DDAH2	Dimethylarginine dimethylaminohydrolase 2	Promotes angiogenesis and associated with invasiveness in lung adenocarcinoma, potential prognostic factor (50)	4.5	4.1	10.6
VPS35L	VPS35 Endosomal Protein Sorting Factor Like	Prognostic (favourable) marker in renal cancer (proteinatlas.org, 14)	7.8	4.0	6.6
NXN	Nucleoredoxin	Increased response to proliferation signals in cancer cells, suggested contribution to colon cancer (51)	3.9	4.5	10.1
EPCAM	Epithelial cell adhesion molecule	**Overexpressed in 55%-75% of ovarian carcinomas** (52); **Risk factor for chemoresistance, a predictive biomarker of chemotherapeutic response** (53); **Associated with poor prognosis in ovarian cancer** (53, 54); **Inclusion in aptamer targetting ovarian cancer improves effectiveness in growth suppression of intraperitoneal ovarian cancer xenografts in mice** (55)	5.9	3.9	4.3
RNASE4	Ribonuclease A family member 4	**Upregulated in ovarian cancer patient serum** (56); Promotes prostate cancer progression through cancer cell proliferation and angiogenesis (57); potential plasma biomarker to predict prostate cancer aggressiveness (57); positive correlation of plasma protein levels with stage, grade and Gleason score in prostate cancer (57)	3.7	4.8	6.4
MLYCD	Malonyl-CoA decarboxylase, mitochondrial	Silencing MLYCD selectively suppresses cancer cell proliferation (58)	3.4	3.8	11.0
TSG101	Tumour susceptibility 101	**Upregulated in ovarian cancer (borderline, low-grade and high-grade), associated with poor prognosis** (59); Up-regulated expression in colorectal cancer (60), gastrointestinal tumour (61), gallbladder cancer (62), hepatocellular carcinoma (63) and papillary thyroid cancer (64)	3.6	3.4	3.9
RPL35A	Ribosomal protein L35a	**Increased expression in ovarian cancer tissues compared to corresponding normal tissue, high expression is associated with short survival, and poor TNM staging** (65)	3.4	9.7	9.6
NEK10	Serine/threonine-protein kinase Nek10	**Mutations present in ovarian cancer (~7%)** and endometrial, pancreatic and skin cancers (66); Mutations associated with breast cancer development (67); Regulates p53 transcriptional activity (68)	3.2	4.4	3.4
CS	Citrate synthase	**Increased expression in malignant ovarian tumours and cell lines compared to benign tumours and normal ovarian surface epithelium** (69); **Potential therapeutic target in ovarian cancer** (69)	4.6	3.2	30.7
SPTAN1	Spectrin alpha, non-erythrocytic 1	**High expression in STIL lesions compared to normal fallopian tissue epithelium and STIC lesions, linked with poor prognosis in ovarian cancer** (70); Prognostic biomarker for colorectal cancer (71); High expression is associated with better survival outcomes in lung cancer (71)	4.0	3.0	38.4
SNRPA	Small nuclear ribonucleoprotein polypeptide A	Higher expression in gastric cancer tumour tissue compared to normal gastric mucosa tissue, high expression indicates poor prognosis in gastric cancer (72); Overexpressed in hepatocellular carcinoma which correlated with poor survival (73)	3.0	3.7	24.4
DSC2	Desmocollin-2	**Overexpressed in ovarian cancer and associated with poor survival** (74); **Identified as a candidate ovarian cancer biomarker from ascites** (75); Prognostic marker in urothelial cancer (unfavourable) and lung cancer (unfavourable) (proteinatlas.org, 14)	3.9	2.9	11.5
ACAD9	Acyl-CoA dehydrogenase family member 9	Included in a proposed prognostic model from colon adenocarcinoma (76); Prognostic marker in renal (favourable), liver (unfavourable) and endometrial cancer (unfavourable) (proteinatlas.org, 14)	3.5	2.7	2.8
MYO1D	Myosin ID	Prognostic marker in renal (unfavourable) and liver cancer (favourable) (proteinatlas.org, 14)	2.7	3.2	4.6
ATP1B1	Sodium/potassium-transporting ATPase subunit beta-1	Expression upregulated in hepatocellular carcinoma (77); Associated with unfavourable prognosis in acute myeloid leukaemia (78); highly expressed in colorectal cancer and associated with metastasis (79)	4.6	2.7	14.3
ZG16B	Zymogen granule protein 16B	**Expression affects malignant behaviour of ovarian cancer, potential therapeutic target** (80); Upregulated in breast cancer and a potential biomarker for breast cancer prognosis (81)	2.8	3.2	2.6
MAOA	Monoamine oxidase A	Elevated levels of expression in non-small cell lung cancer at the advanced stage, expression correlates with later stage and lymph node metastasis (82); Tissue proteins levels correlated with serum prostate specific antigen, may contribute to the growth of prostate cancer (83)	2.9	2.7	2.6
MUC4	Mucin 4, Cell Surface Associated	**Overexpressed in ovarian cancer cells compared control cell lines** (84); **High expression in early and late ovarian cancer tumour samples, MUC4 had the highest prevalence compared to other mucins in advanced ovarian cancer** (85)	3.0	3.7	2.6
CFAP52	Cilia- and flagella-associated protein 52	Increased in p53 lesions compared to incidental STIC lesions (86); Enhanced in the fallopian tube and **prognostic marker (favourable) in ovarian cancer** (proteinatlas.org, 14)	5.9	11.1	2.5
NLRC4	NLR family CARD domain containing 4	**Enriched in ovarian tumour tissue** (87); Low expression in colorectal cancer tissues and cell lines and expression significantly associated with lymph node metastasis (88)	2.5	2.9	2.8
TMED7	Transmembrane p24 trafficking protein 7	Increased expression in head-neck squamous cell carcinoma compared to normal tissue (89)	2.5	5.1	3.0

CALM1	Calmodulin 1	**Elevated in HGSOC tissues from patients who responded to chemotherapy compared to those who did not, potential predictive biomarker for chemotherapy response** (90); Potential diagnostic and prognostic biomarker in cancer, contributes to the activation of various cancer-related pathways such as WNT and MAPK (91); Overexpressed in oesophageal cancer and associated with stage and poor prognosis (92)	7.9	3.8	2.4
SDCBP	Syndecan binding protein	**SDCBP antibodies are produced in ovarian cancer patients, SDCBP-specific IgG4 reduces tumour growth in a HGSOC mouse model (subcut OVCAR3)** (93); Promotes proliferation, migration and invasion (93); Associated with unfavourable prognosis in breast (94) and colorectal cancer (95); Potential therapeutic target for cancer metastases (96)	4.7	2.4	12.4
WDR13	WD repeat domain 13	**Prognostic (favourable) in ovarian cancer**, glioma and renal cancer (proteinatlas.org, 14); **Downregulated in ascites which stimulate invasion of OV-90 cells** (97)	2.4	2.8	2.5
ANXA13	Annexin A13	**Downregulated in ovarian cancer (OC), genetic variations may be correlated with OC tumorigenesis and progression** (98); Promotes tumour cell invasion *in vitro* , associated with metastasis and poor survival in colorectal cancer (99); Increased expression in cholangiocarcinoma tissue which negatively correlated with kidney renal clear cell carcinoma stage and negatively correlated with immune infiltration in a range of cancer types (100)	4.7	2.4	6.3
HDGFL3	HDGF like 3	Increased expression in hepatocellular carcinoma tissue, plays an essential role in HCC pathogenesis and contributes to chemoresistance (101); Prognostic in glioma (favourable), pancreatic cancer (favourable), lung cancer (unfavourable) and stomach cancer (unfavourable) (proteinatlas.org, 14)	3.4	4.4	2.4
PCBP1	Poly(rC) binding protein 1	**Downregulation associated with ovarian cancer tumour progression** (102); Possibly interacts with positive regulatory region of BRCA1 promotor (103); Mediates drug resistance in colorectal cancer and a possible drug target (104)	2.5	2.9	2.4
RPL15	Ribosomal protein L15	**Increased expression in blood exosomes of ovarian cancer patients which correlated with survival** (105); Upregulated in gastric cancer and associated with cell proliferation (106); Downregulation in pancreatic ductal cancer associated with progression and poor survival (107)	2.8	4.1	2.3
ENAM	Enamelin	Prognostic (favourable) in renal cancer (proteinatlas.org, 14); Associated with survival in clear cell renal cell cancer (ccRCC), potentially prognostic as part of a seven gene signature (108); Inhibits proliferation in ccRCC and associated with immune cell infiltration (109)	2.3	3.7	3.9
PREPL	Prolyl endopeptidase-like	Prognostic (favourable) in kidney renal clear cell carcinoma (proteinatlas.org, 14)	2.7	3.0	2.3
CAVIN2	Caveolae associated protein 2	**High expression in STIL lesions compared to FTE and STIC lesions, absent in STIC lesions** (70); Linked with prognosis in renal cancer (favourable) and stomach cancer (unfavourable) (70); Strong staining (IHC) in endometroid carcinoma of the ovary (70)	3.7	2.4	2.3
PLOD2	Procollagen-lysine,2-oxoglutarate 5-dioxygenase 2	**Highly expressed in a wide variety of tumours, including ovarian cancer (OC)** (110) **Associated with immune cell infiltration, including in OC** (110)	2.3	3.6	6.3
EGFR	Epidermal growth factor receptor	**Established drug target in epithelial ovarian cancer (OC)** (111, 112); **Overexpressed in 30-98% of epithelial OC** (112); Increased in normal BRCA1-mutated tissues compared to controls, BRCA1 knockdown activated EGFR in ovarian cancer cells (113); Established drug target across multiple cancer types (114)	2.3	3.1	4.3
VPS18	VPS18 core subunit of CORVET and HOPS complexes	**VPS18 gene identified as a risk factor in epithelial ovarian cancer (EOC) and part of a ubiquitin-related mRNA signature predictive of worse prognosis in EOC** (115); Prognostic in renal (favourable) and cervical (unfavourable) cancer (proteinatlas.org, 14)	2.3	2.5	3.5
PEF1	Peflin	Prognostic in pancreatic (favourable), pancreatic (favourable) and liver cancer (unfavourable) (proteinatlas.org, 14)	2.2	2.6	2.2
NCKAP1	NCK associated protein 1	Linked to MHC-I expression: knockdown increased MHC-I expression and induced immunogenic death of breast cancer cells (116); Differentially expressed and correlated with prognosis in a wide range of cancers (117)	2.2	5.7	6.5
TJP1	Tight junction protein 1	Reduced expression in lung cancer cell lines inhibits migration and invasion and potential membrane drug target for lung cancer (118); Highly expressed in pancreatic cancer and low expression associated with better prognosis/survival (118); Plays multiple roles in cancer, including cancer growth and motility and angiogenesis promotion (119)	3.3	2.2	13.2
ZBTB8OS	Zinc finger and BTB domain containing 8 opposite strand	**ZBTB8OS-AC090627.1 fusion transcript highly associated with worse survival in high-grade serous ovarian cancer (HGSOC)** (120); Prognostic (unfavourable) in liver cancer (proteinatlas.org, 14)	3.0	2.3	2.2
GSTM5	Glutathione S-transferase mu 5	**Decreased in ovarian cancer (OC) compared to normal ovary, positively correlated with OC prognosis and negatively correlated with stemness** (121); Reduced expression in lung cancer, low expression associated with poor survival (122); Reduced expression in bladder cancer, identified as potential biomarker (123)	2.2	2.2	2.8
PFDN6	Prefoldin subunit 6	**High expression associated with improved survival in ovarian cancer** (124); Increased expression in colorectal cancer and associated with colorectal cancer progression (125)	3.4	2.1	5.7
ARF6	ADP ribosylation factor 6	**Essential for Pten-loss induced increased invasiveness in high-grade serous ovarian cancer (HGSOC), potential therapeutic target in PTEN-depleted HGSOC** (126); **High levels predictive of poor survival in HGSOC when combined with AGAP1** (126)	2.5	2.1	4.8
HDAC2	Histone Deacetylase 2	**Higher protein expression in ovarian serous and mucinous cancer tissue compared to normal tissues, increased protein expression was found in 72% of 18 ovarian cancer samples** (127); Prognostic marker in breast (unfavourable), liver hepatocellular (unfavourable), chromophobe renal cell (unfavourable) and kidney renal cell cancer (favourable) (proteinatlas.org, 14)	2.5	3.0	2.1
GYS1	Glycogen synthase 1	High expression associated with poor survival in triple-negative breast cancer, high expression associated with ki-67-high primary breast tumours, knockdown impairs breast cancer proliferation (128)	11.6	2.1	3.0

ERICH3	Glutamate rich 3	Differentially expressed between homologous recombination repair (HRR)-related phenotypes in breast cancer and part of a prognosis risk model (129)	6.1	16.8	2.1
RTN4	Reticulon 4	**High expression correlates with poor survival in ovarian cancer**, as well as breast, lung, cervical and kidney cancer (130); Knockdown inhibits breast cancer proliferation, migration and invasion *in vitro* and inhibits xenograft tumour growth in mice (131)	4.6	2.1	2.6
PSME3	Proteasome activator subunit 3	Found to be differentially expressed in 15/16 cancers analysed, with overexpression in 12/16 (132); Induces epithelial-to-mesenchymal transition (EMT), migration and invasion of MDA-MB-231 (breast cancer) cells (133)	3.0	2.8	2.1
ARHGAP1	Rho GTPase activating protein 1	Promotes hepatocellular tumour progression and elevated expression linked to poor survival (134); Overexpressed in cervical cancer cells (135)	4.6	3.4	2.0
ANO6	Anoctamin 6	**Part of a ferroptosis-related prognostic gene signature for ovarian cancer** (136); Differential expression in a wide range of cancers compared to control tissue, with **lower expression in ovarian serous cystadenocarcinoma** (137); Overexpression predicts poor survival in breast cancer (138)	4.0	2.0	3.4
MTMR2	Myotubularin related protein 2	Promotes invasion and metastasis in gastric cancer, potential prognostic marker (139); Prognostic marker in liver (unfavourable), lung (unfavourable) and renal cancer (favourable) (proteinatlas.org, 14)	2.0	2.5	6.6
HMGB3	High mobility group box 3	**Overexpressed in high-grade serous ovarian cancer (HGSOC) tissue, high levels indicate reduced survival and increased resistance to PARP inhibitors in cell lines** (140); **Promotes proliferation, cancer stemness and metastasis in ovarian cancer cell lines, and promotes tumour xenograft growth *in vivo*** (141); **Potential target to reduce chemoresistance in ovarian cancer** (142)	2.0	4.1	10.5
PODXL	Podocalyxin like	**Altered cellular type and localisation of staining in high-grade serous ovarian cancer (HGSOC) tissues compared to benign ovarian tissue, expressed in ovarian cancer cell lines with higher expression in HGSOC-like lines, promotes formation of compact spheroids with resistance to chemotherapeutics** (143); Upregulated in various cancer types and often indicates poor prognosis (144)	2.7	2.0	3.1
CNN1	Calponin-1	**Downregulated in high-grade ovarian cancer (HGSOC) in ovaries and fallopian tube compared to normal tissue, downregulation potentially essential for HGSOC metastasis** (145); Lower expression in cancer compared to normal tissue in most cancers, but expression increases as tumours develop and high levels associated with poor prognosis (146)	2.0	9.0	2.2
TNS1	Tensin 1	More frequently present in metastatic gastric tumours and expression associated with poor prognosis (147); Increased levels in non-small cell lung cancer (NSCLC) and expression correlates with poor prognosis, promotes growth and metastasis of NSCLC cells (148)	2.0	2.3	5.4
NUDT16L1	Nudix hydrolase 16 like 1	Overexpressed in colorectal cancer tissues and overexpression promotes tumour growth in a mouse model of colon cancer (149)	2.0	2.0	2.0

Number	Reference
1	Dufresne, J., Bowden, P., Thavarajah, T. *et al.* The plasma peptides of ovarian cancer. *Clin Proteom* **15**, 41 (2018). https://doi.org/10.1186/s12014-018-9215-z
2	Mendonça JB, de Araújo NS, Sassaro TF, Alberto AVP, Carrossini N, Fernandes PV, Costa MA, Guimarães ACR, Degrave WMS, Waghabi MC. Next Generation of Ovarian Cancer Detection Using Aptamers. Int J Mol Sci. 2023 Mar 28;24(7):6315. doi: 10.3390/ijms24076315
3	Mala U, Baral TK, Somasundaram K. Integrative analysis of cell adhesion molecules in glioblastoma identified prostaglandin F2 receptor inhibitor (PTGFRN) as an essential gene. BMC Cancer. 2022 Jun 11;22(1):642. doi: 10.1186/s12885-022-09682-2
4	Fassad MR, Rumman N, Junger K, Patel MP, Thompson J, Goggin P, Ueffing M, Beyer T, Boldt K, Lucas JS, Mitchison HM. Defective airway intraflagellar transport underlies a combined motile and primary ciliopathy syndrome caused by IFT74 mutations. Hum Mol Genet. 2023 Oct 17;32(21):3090-3104. doi: 10.1093/hmg/ddad132
5	Egeberg DL, Lethan M, Manguso R, Schneider L, Awan A, Jørgensen TS, Byskov AG, Pedersen LB, Christensen ST. Primary cilia and aberrant cell signaling in epithelial ovarian cancer. Cilia. 2012 Aug 10;1(1):15. doi: 10.1186/2046-2530-1-15
6	Fabbri L, Bost F, Mazure NM. Primary Cilium in Cancer Hallmarks. Int J Mol Sci. 2019 Mar 16;20(6):1336. doi: 10.3390/ijms20061336
7	Casalou C, Faustino A, Silva F, Ferreira IC, Vaqueirinho D, Ferreira A, Castanheira P, Barona T, Ramalho JS, Serpa J, Félix A, Barral DC. Arl13b Regulates Breast Cancer Cell Migration and Invasion by Controlling Integrin-Mediated Signaling. Cancers (Basel). 2019 Sep 29;11(10):1461. doi: 10.3390/cancers11101461
8	Shao J, Xu L, Chen L, Lu Q, Xie X, Shi W, Xiong H, Shi C, Huang X, Mei J, Rao H, Lu H, Lu N, Luo S. Arl13b Promotes Gastric Tumorigenesis by Regulating Smo Trafficking and Activation of the Hedgehog Signaling Pathway. Cancer Res. 2017 Aug 1;77(15):4000-4013. doi: 10.1158/0008-5472.CAN-16-2461
9	Casalou C, Ferreira A, Barral DC. The Role of ARF Family Proteins and Their Regulators and Effectors in Cancer Progression: A Therapeutic Perspective. Front Cell Dev Biol. 2020 Apr 21;8:217. doi: 10.3389/fcell.2020.00217
10	Go G, Lee SH. The Cellular Prion Protein: A Promising Therapeutic Target for Cancer. Int J Mol Sci. 2020 Dec 2;21(23):9208. doi: 10.3390/ijms21239208
11	Ding M, Chen Y, Lang Y, Cui L. The Role of Cellular Prion Protein in Cancer Biology: A Potential Therapeutic Target. Front Oncol. 2021 Sep 14;11:742949. doi: 10.3389/fonc.2021.742949
12	Déry MA, Jodoin J, Ursini-Siegel J, Aleynikova O, Ferrario C, Hassan S, Basik M, LeBlanc AC. Endoplasmic reticulum stress induces PRNP prion protein gene expression in breast cancer. Breast Cancer Res. 2013 Mar 12;15(2):R22. doi: 10.1186/bcr3398
13	Mouillet-Richard S, Ghazi A, Laurent-Puig P. The Cellular Prion Protein and the Hallmarks of Cancer. Cancers (Basel). 2021 Oct 8;13(19):5032. doi: 10.3390/cancers13195032
14	Uhlen M, Zhang C, Lee S, Sjöstedt E, Fagerberg L, Bidkhori G, Benfeitas R, Arif M, Liu Z, Edfors F, Sanli K, von Feilitzen K, Oksvold P, Lundberg E, Hober S, Nilsson P, Mattsson J, Schwenk JM, Brunnström H, Glimelius B, Sjöblom T, Edqvist PH, Djureinovic D, Micke P, Lindskog C, Mardinoglu A, Ponten F. A pathology atlas of the human cancer transcriptome. Science. 2017 Aug 18;357(6352):eaan2507. doi: 10.1126/science.aan2507; available at v24.proteinatlas.org
15	Sawaki K, Kanda M, Umeda S, Miwa T, Tanaka C, Kobayashi D, Hayashi M, Yamada S, Nakayama G, Omae K, Koike M, Kodera Y. Level of Melanotransferrin in Tissue and Sera Serves as a Prognostic Marker of Gastric Cancer. Anticancer Res. 2019 Nov;39(11):6125-6133. doi: 10.21873/anticanres.13820
16	Shin J, Kim HJ, Kim G, Song M, Woo SJ, Lee ST, Kim H, Lee C. Discovery of melanotransferrin as a serological marker of colorectal cancer by secretome analysis and quantitative proteomics. J Proteome Res. 2014 Nov 7;13(11):4919-31. doi: 10.1021/pr500790f
17	Eriksson J, Le Joncour V, Nummela P, Jahkola T, Virolainen S, Laakkonen P, Saksela O, Hölttä E. Gene expression analyses of primary melanomas reveal CTHRC1 as an important player in melanoma progression. Oncotarget. 2016 Mar 22;7(12):15065-92. doi: 10.18632/oncotarget.7604

18	Wang Y, Li L, Zhang X, Zhao X. Long non-coding RNA OIP5-AS1 suppresses microRNA-92a to augment proliferation and metastasis of ovarian cancer cells through upregulating ITGA6. J Ovarian Res. 2022 Feb 16;15(1):25. doi: 10.1186/s13048-021-00937-3
19	Schröfelbauer B, Kimes PK, Hauke P, Reid CE, Shao K, Hill SJ, Irizarry R, Hahn WC. Discovery of antibodies and cognate surface targets for ovarian cancer by surface profiling. Proc Natl Acad Sci U S A. 2023 Jan 3;120(1):e2206751120. doi: 10.1073/pnas.2206751120
20	Gambelli A, Nespolo A, Rampioni Vinciguerra GL, Pivetta E, Pellarin I, Nicoloso MS, Scapin C, Stefenatti L, Segatto I, Favero A, D'Andrea S, Mucignat MT, Bartoletti M, Lucia E, Schiappacassi M, Spessotto P, Canzonieri V, Giorda G, Puglisi F, Vecchione A, Belletti B, Sonego M, Baldassarre G. Platinum-induced upregulation of ITGA6 promotes chemoresistance and spreading in ovarian cancer. EMBO Mol Med. 2024 May;16(5):1162-1192. doi: 10.1038/s44321-024-00069-3
21	Cai X, Lin J, Liu L, Zheng J, Liu Q, Ji L, Sun Y. A novel TCGA-validated programmed cell-death-related signature of ovarian cancer. BMC Cancer. 2024 Apr 23;24(1):515. doi: 10.1186/s12885-024-12245-2
22	Liu Y, Cao H, Zhao Y, Shan L, Lan S. Fisetin-induced cell death in human ovarian cancer cell lines via zbp1-mediated necroptosis. J Ovarian Res. 2022 May 10;15(1):57. doi: 10.1186/s13048-022-00984-4
23	Bruand M, Barras D, Mina M, Ghisoni E, Morotti M, Lanitis E, Fahr N, Desbuisson M, Grimm A, Zhang H, Chong C, Dagher J, Chee S, Tsianou T, Dorier J, Stevenson BJ, Iseli C, Ronet C, Bobisse S, Genolet R, Walton J, Bassani-Sternberg M, Kandalaft LE, Ren B, McNeish I, Swisher E, Harari A, Delorenzi M, Ciriello G, Irving M, Rusakiewicz S, Foukas PG, Martinon F, Dangaj Laniti D, Coukos G. Cell-autonomous inflammation of BRCA1-deficient ovarian cancers drives both tumor-intrinsic immunoreactivity and immune resistance via STING. Cell Rep. 2021 Jul 20;36(3):109412. doi: 10.1016/j.celrep.2021.109412
24	Baik JY, Liu Z, Jiao D, Kwon HJ, Yan J, Kadigamuwa C, Choe M, Lake R, Kruhlak M, Tandon M, Cai Z, Choksi S, Liu ZG. ZBP1 not RIPK1 mediates tumor necroptosis in breast cancer. Nat Commun. 2021 May 11;12(1):2666. doi: 10.1038/s41467-021-23004-3
25	Noriega-Rivera R, Rivera-Serrano M, Rabelo-Fernandez RJ, Pérez-Santiago J, Valiyeva F, Vivas-Mejía PE. Upregulation of the Long Noncoding RNA CASC10 Promotes Cisplatin Resistance in High-Grade Serous Ovarian Cancer. Int J Mol Sci. 2022 Jul 13;23(14):7737. doi: 10.3390/ijms23147737
26	Bignotti E, Tassi RA, Calza S, Ravaggi A, Bandiera E, Rossi E, Donzelli C, Pasinetti B, Pecorelli S, Santin AD. Gene expression profile of ovarian serous papillary carcinomas: identification of metastasis-associated genes. Am J Obstet Gynecol. 2007 Mar;196(3):245.e1-11. doi: 10.1016/j.ajog.2006.10.874
27	Hu X, Chen M, Ruan Q, Shi C, Pan J, Luo L. Comprehensive Analysis of PDLIM3 Expression Profile, Prognostic Value, and Correlations with Immune Infiltrates in Gastric Cancer. J Immunol Res. 2022 May 12;2022:2039447. doi: 10.1155/2022/2039447
28	Zhang D, Wang F, Pang Y, Zhao E, Zhu S, Chen F, Cui H. ALG2 regulates glioblastoma cell proliferation, migration and tumorigenicity. Biochem Biophys Res Commun. 2017 Apr 29;486(2):300-306. doi: 10.1016/j.bbrc.2017.03.032
29	la Cour JM, Høj BR, Mollerup J, Simon R, Sauter G, Berchtold MW. The apoptosis linked gene ALG-2 is dysregulated in tumors of various origin and contributes to cancer cell viability. Mol Oncol. 2008 Apr;1(4):431-9. doi: 10.1016/j.molonc.2007.08.002
30	Tchagang AB, Tewfik AH, DeRycke MS, Skubitz KM, Skubitz AP. Early detection of ovarian cancer using group biomarkers. Mol Cancer Ther. 2008 Jan;7(1):27-37. doi: 10.1158/1535-7163.MCT-07-0565
31	Reinartz S, Finkernagel F, Adhikary T, Rohnalter V, Schumann T, Schober Y, Nockher WA, Nist A, Stiewe T, Jansen JM, Wagner U, Müller-Brüsselbach S, Müller R. A transcriptome-based global map of signaling pathways in the ovarian cancer microenvironment associated with clinical outcome. Genome Biol. 2016 May 23;17(1):108. doi: 10.1186/s13059-016-0956-6
32	Abreu RDS, Antunes D, Moreira ADS, Passetti F, Mendonça JB, de Araújo NS, Sassaro TF, Alberto AVP, Carrossini N, Fernandes PV, Costa MA, Guimarães ACR, Degrave WMS, Waghabi MC. Next Generation of Ovarian Cancer Detection Using Aptamers. Int J Mol Sci. 2023 Mar 28;24(7):6315. doi: 10.3390/ijms24076315

33	Medrano M, Communal L, Brown KR, Iwanicki M, Normand J, Paterson J, Sircoulomb F, Krzyzanowski P, Novak M, Doodnauth SA, Saiz FS, Cullis J, Al-Awar R, Neel BG, McPherson J, Drapkin R, Ailles L, Mes-Massons AM, Rottapel R. Interrogation of Functional Cell-Surface Markers Identifies CD151 Dependency in High-Grade Serous Ovarian Cancer. Cell Rep. 2017 Mar 7;18(10):2343-2358. doi: 10.1016/j.celrep.2017.02.028
34	Zhang H, Song Q, Shang K, Li Y, Jiang L, Yang L. Tspan protein family: focusing on the occurrence, progression, and treatment of cancer. Cell Death Discov. 2024 Apr 22;10(1):187. doi: 10.1038/s41420-024-01961-0
35	Sidahmed-Adrar N, Ottavi JF, Benzoubir N, Ait Saadi T, Bou Saleh M, Mauduit P, Guettier C, Desterke C, Le Naour F. Tspan15 Is a New Stemness-Related Marker in Hepatocellular Carcinoma. Proteomics. 2019 Nov;19(21-22):e1900025. doi: 10.1002/pmic.201900025
36	Ryoo ZY, Jung BK, Lee SR, Kim MO, Kim SH, Kim HJ, Ahn JY, Lee TH, Cho YH, Park JH, Kim JK. Neoplastic transformation and tumorigenesis associated with overexpression of IMUP-1 and IMUP-2 genes in cultured NIH/3T3 mouse fibroblasts. Biochem Biophys Res Commun. 2006 Oct 27;349(3):995-1002. doi: 10.1016/j.bbrc.2006.08.137
37	Luo Q, Pan Y, Fu Q, Zhang X, Zhou S, Yu P, Tian H, Liu P, Chen S, Zhang H, Qin T. Immortalization-upregulated protein promotes pancreatic cancer progression by regulating NPM1/FHL1-mediated cell-cycle-checkpoint protein activity. Cell Biol Toxicol. 2023 Oct;39(5):2069-2087. doi: 10.1007/s10565-022-09695-4
38	Wei R, Qi G, Zeng Z, Shen N, Wang Z, Shen H, Gao L, Song C, Ma W, Wang C. IMUP and GPRC5A: two newly identified risk score indicators in pancreatic ductal adenocarcinoma. Cancer Cell Int. 2021 Nov 24;21(1):620. doi: 10.1186/s12935-021-02324-w
39	Zhao Y, Niu LT, Hu LJ, Lv M. Comprehensive analysis of ECHDC3 as a potential biomarker and therapeutic target for acute myeloid leukemia: Bioinformatic analysis and experimental verification. Front Oncol. 2022 Sep 12;12:947492. doi: 10.3389/fonc.2022.947492
40	Xu Q, Deng B, Li M, Chen Y, Zhuan L. circRNA-UBAP2 promotes the proliferation and inhibits apoptosis of ovarian cancer though miR-382-5p/PRPF8 axis. J Ovarian Res. 2020 Jul 20;13(1):81. doi: 10.1186/s13048-020-00685-w
41	Cao D, Xue J, Huang G, An J, An W. The role of splicing factor PRPF8 in breast cancer. Technol Health Care. 2022;30(S1):293-301. doi: 10.3233/THC-THC228028
42	Liang Y, Song X, Li Y, Su P, Han D, Ma T, Guo R, Chen B, Zhao W, Sang Y, Zhang N, Li X, Zhang H, Liu Y, Duan Y, Wang L, Yang Q. circKDM4C suppresses tumor progression and attenuates doxorubicin resistance by regulating miR-548p/PBLD axis in breast cancer. Oncogene. 2019 Oct;38(42):6850-6866. doi: 10.1038/s41388-019-0926-z
43	Mei J, Pan L, Huang M, Bao D, Gao H, Wang D. DDOST is associated with tumor immunosuppressive microenvironment in cervical cancer. Discov Oncol. 2024 Mar 9;15(1):69. doi: 10.1007/s12672-024-00927-z
44	Hapke S, Kessler H, Luber B, Benge A, Hutzler P, Höfler H, Schmitt M, Reuning U. Ovarian cancer cell proliferation and motility is induced by engagement of integrin alpha(v)beta3/Vitronectin interaction. Biol Chem. 2003 Jul;384(7):1073-83. doi: 10.1515/BC.2003.120
45	Sandow JJ, Rainczuk A, Infusini G, Makanji M, Bilandzic M, Wilson AL, Fairweather N, Stanton PG, Garama D, Gough D, Jobling TW, Webb AI, Stephens AN. Discovery and Validation of Novel Protein Biomarkers in Ovarian Cancer Patient Urine. Proteomics Clin Appl. 2018 May;12(3):e1700135. doi: 10.1002/prca.201700135
46	Ni M, Zhou J, Zhu Z, Yuan J, Gong W, Zhu J, Zheng Z, Zhao H. A Novel Classifier Based on Urinary Proteomics for Distinguishing Between Benign and Malignant Ovarian Tumors. Front Cell Dev Biol. 2021 Aug 30;9:712196. doi: 10.3389/fcell.2021.712196
47	Zhang J, Huang S, Quan L, Meng Q, Wang H, Wang J, Chen J. Determination of Potential Therapeutic Targets and Prognostic Markers of Ovarian Cancer by Bioinformatics Analysis. Biomed Res Int. 2021 Mar 19;2021:8883800. doi: 10.1155/2021/8883800
48	Hameed Y, Usman M, Liang S, Ejaz S. Novel diagnostic and prognostic biomarkers of colorectal cancer: Capable to overcome the heterogeneity-specific barrier and valid for global applications. PLoS One. 2021 Sep 2;16(9):e0256020. doi: 10.1371/journal.pone.0256020

49	Sang Q, Li X, Wang H, Wang H, Zhang S, Feng R, Xu Y, Li Q, Zhao X, Xing Q, Jin L, He L, Wang L. Quantitative methylation level of the EPHX1 promoter in peripheral blood DNA is associated with polycystic ovary syndrome. PLoS One. 2014 Feb 5;9(2):e88013. doi: 10.1371/journal.pone.0088013
50	Shiozawa T, Iyama S, Toshima S, Sakata A, Usui S, Minami Y, Sato Y, Hizawa N, Noguchi M. Dimethylarginine dimethylaminohydrolase 2 promotes tumor angiogenesis in lung adenocarcinoma. Virchows Arch. 2016 Feb;468(2):179-90. doi: 10.1007/s00428-015-1863-z
51	Idelfonso-García OG, Alarcón-Sánchez BR, Vásquez-Garzón VR, Baltiérrez-Hoyos R, Villa-Treviño S, Muriel P, Serrano H, Pérez-Carreón JI, Arellanes-Robledo J. Is Nucleoredoxin a Master Regulator of Cellular Redox Homeostasis? Its Implication in Different Pathologies. Antioxidants (Basel). 2022 Mar 30;11(4):670. doi: 10.3390/antiox11040670
52	Spizzo G, Fong D, Wurm M, Ensinger C, Obrist P, Hofer C, Mazzoleni G, Gastl G, Went P. EpCAM expression in primary tumour tissues and metastases: an immunohistochemical analysis. J Clin Pathol. 2011 May;64(5):415-20. doi: 10.1136/jcp.2011.090274
53	Tayama S, Motohara T, Narantuya D, Li C, Fujimoto K, Sakaguchi I, Tashiro H, Saya H, Nagano O, Katabuchi H. The impact of EpCAM expression on response to chemotherapy and clinical outcomes in patients with epithelial ovarian cancer. Oncotarget. 2017 Jul 4;8(27):44312-44325. doi: 10.18632/oncotarget.17871
54	Spizzo G, Went P, Dirnhofer S, Obrist P, Moch H, Baeuerle PA, Mueller-Holzner E, Marth C, Gastl G, Zeimet AG. Overexpression of epithelial cell adhesion molecule (Ep-CAM) is an independent prognostic marker for reduced survival of patients with epithelial ovarian cancer. Gynecol Oncol. 2006 Nov;103(2):483-8. doi: 10.1016/j.ygyno.2006.03.035
55	Zheng J, Zhao S, Yu X, Huang S, Liu HY. Simultaneous targeting of CD44 and EpCAM with a bispecific aptamer effectively inhibits intraperitoneal ovarian cancer growth. Theranostics. 2017 Mar 23;7(5):1373-1388. doi: 10.7150/thno.17826
56	Timms JF, Arslan-Low E, Kabir M, Worthington J, Camuzeaux S, Sinclair J, Szaub J, Afrough B, Podust VN, Fourkala EO, Cubizolles M, Kronenberg F, Fung ET, Gentry-Maharaj A, Menon U, Jacobs I. Discovery of serum biomarkers of ovarian cancer using complementary proteomic profiling strategies. Proteomics Clin Appl. 2014 Dec;8(11-12):982-93. doi: 10.1002/prca.201400063
57	Vanli N, Sheng J, Li S, Xu Z, Hu GF. Ribonuclease 4 is associated with aggressiveness and progression of prostate cancer. Commun Biol. 2022 Jun 25;5(1):625. doi: 10.1038/s42003-022-03597-1
58	Yizhak K, Gaude E, Le Dévédec S, Waldman YY, Stein GY, van de Water B, Frezza C, Ruppin E. Phenotype-based cell-specific metabolic modeling reveals metabolic liabilities of cancer. Elife. 2014 Nov 21;3:e03641. doi: 10.7554/eLife.03641
59	Young TW, Rosen DG, Mei FC, Li N, Liu J, Wang XF, Cheng X. Up-regulation of tumor susceptibility gene 101 conveys poor prognosis through suppression of p21 expression in ovarian cancer. Clin Cancer Res. 2007 Jul 1;13(13):3848-54. doi: 10.1158/1078-0432.CCR-07-0337
60	Ma XR, Edmund Sim UH, Pauline B, Patricia L, Rahman J. Overexpression of WNT2 and TSG101 genes in
61	Koon N, Schneider-Stock R, Sarlomo-Rikala M, Lasota J, Smolkin M, Petroni G, Zaika A, Boltze C, Meyer F, Andersson L, Knuutila S, Miettinen M, El-Rifai W. Molecular targets for tumour progression in gastrointestinal stromal tumours. Gut. 2004 Feb;53(2):235-40. doi: 10.1136/gut.2003.021238
62	Liu DC, Yang ZL, Jiang S. Identification of PEG10 and TSG101 as carcinogenesis, progression, and poor-prognosis related biomarkers for gallbladder adenocarcinoma. Pathol Oncol Res. 2011 Dec;17(4):859-66. doi: 10.1007/s12253-011-9394-7
63	Liu Z, Tian Z, Cao K, Zhang B, Wen Q, Zhou X, Yang W, Wang T, Shi H, Wang R. TSG101 promotes the proliferation, migration and invasion of hepatocellular carcinoma cells by regulating the PEG10. J Cell Mol Med. 2019 Jan;23(1):70-82. doi: 10.1111/jcmm.13878
64	Liu RT, Huang CC, You HL, Chou FF, Hu CC, Chao FP, Chen CM, Cheng JT. Overexpression of tumor susceptibility gene TSG101 in human papillary thyroid carcinomas. Oncogene. 2002 Jul 18;21(31):4830-7. doi: 10.1038/sj.onc.1205612
65	Wu H, Xia L, Sun L, Li D, Liu X, Song H, Sheng J, Wang K, Feng Q. RPL35A drives ovarian cancer progression by promoting the binding of YY1 to CTCF promoter. J Cell Mol Med. 2024 Mar;28(6):e18115. doi: 10.1111/jcmm.18115

66	Nguyen K, Boehling J, Tran MN, Cheng T, Rivera A, Collins-Burow BM, Lee SB, Drewry DH, Burow ME. NEK Family Review and Correlations with Patient Survival Outcomes in Various Cancer Types. Cancers (Basel). 2023 Mar 30;15(7):2067. doi: 10.3390/cancers15072067
67	Ramirez-Ardila DE, Ruigrok-Ritstier K, Helmijr JC, Look MP, van Laere S, Dirix L, Berns EM, Jansen MP. LRG1 mRNA expression in breast cancer associates with PIK3CA genotype and with aromatase inhibitor therapy outcome. Mol Oncol. 2016 Oct;10(8):1363-73. doi: 10.1016/j.molonc.2016.07.004
68	Haider N, Dutt P, van de Kooij B, Ho J, Palomero L, Pujana MA, Yaffe M, Stambolic V. NEK10 tyrosine phosphorylates p53 and controls its transcriptional activity. Oncogene. 2020 Jul;39(30):5252-5266. doi: 10.1038/s41388-020-1361-x
69	Chen L, Liu T, Zhou J, Wang Y, Wang X, Di W, Zhang S. Citrate synthase expression affects tumor phenotype and drug resistance in human ovarian carcinoma. PLoS One. 2014 Dec 29;9(12):e115708. doi: 10.1371/journal.pone.0115708
70	Wisztorski M, Aboulouard S, Roussel L, Duhamel M, Saudemont P, Cardon T, Narducci F, Robin YM, Lemaire AS, Bertin D, Hajjaji N, Kobeissy F, Leblanc E, Fournier I, Salzet M. Fallopian tube lesions as potential precursors of early ovarian cancer: a comprehensive proteomic analysis. Cell Death Dis. 2023 Sep 30;14(9):644. doi: 10.1038/s41419-023-06165-5
71	Schrecker C, Behrens S, Schönherr R, Ackermann A, Pauli D, Plotz G, Zeuzem S, Brieger A. SPTAN1 Expression Predicts Treatment and Survival Outcomes in Colorectal Cancer. Cancers (Basel). 2021 Jul 20;13(14):3638. doi: 10.3390/cancers13143638
72	Dou N, Yang D, Yu S, Wu B, Gao Y, Li Y. SNRPA enhances tumour cell growth in gastric cancer through modulating NGF expression. Cell Prolif. 2018 Oct;51(5):e12484. doi: 10.1111/cpr.12484
73	Mo Z, Li R, Cao C, Li Y, Zheng S, Wu R, Xue J, Hu J, Meng H, Zhai H, Huang W, Zheng F, Zhou B. Splicing factor SNRPA associated with microvascular invasion promotes hepatocellular carcinoma metastasis through activating NOTCH1/Snail pathway and is mediated by circSEC62/miR-625-5p axis. Environ Toxicol. 2023 May;38(5):1022-1037. doi: 10.1002/tox.23745
74	Su R, Jin C, Jin C, Kuang M, Xiang J. Identification of hub genes in key hallmarks of ovarian cancer via bioinformatics analysis. Transl Cancer Res. 2021 Feb;10(2):827-841. doi: 10.21037/tcr-20-2604
75	Kuk C, Kulasingam V, Gunawardana CG, Smith CR, Batruch I, Diamandis EP. Mining the ovarian cancer ascites proteome for potential ovarian cancer biomarkers. Mol Cell Proteomics. 2009 Apr;8(4):661-9. doi: 10.1074/mcp.M800313-MCP200
76	Lv L, Huang Y, Li Q, Wu Y, Zheng L. A Comprehensive Prognostic Model for Colon Adenocarcinoma Depending on Nuclear-Mitochondrial-Related Genes. Technol Cancer Res Treat. 2024 Jan-Dec;23:15330338241258570. doi: 10.1177/15330338241258570
77	Lu S, Cai S, Peng X, Cheng R, Zhang Y. Integrative Transcriptomic, Proteomic and Functional Analysis Reveals ATP1B3 as a Diagnostic and Potential Therapeutic Target in Hepatocellular Carcinoma. Front Immunol. 2021 Apr 2;12:636614. doi: 10.3389/fimmu.2021.636614
78	Shi JL, Fu L, Ang Q, Wang GJ, Zhu J, Wang WD. Overexpression of ATP1B1 predicts an adverse prognosis in cytogenetically normal acute myeloid leukemia. Oncotarget. 2016 Jan 19;7(3):2585-95. doi: 10.18632/oncotarget.6226
79	Baker Bechmann M, Rotoli D, Morales M, Maeso Mdel C, García Mdel P, Ávila J, Mobasheri A, Martín-Vasallo P. Na,K-ATPase Isozymes in Colorectal Cancer and Liver Metastases. Front Physiol. 2016 Jan 29;7:9. doi: 10.3389/fphys.2016.00009
80	Kim YJ, Jiang F, Park J, Jeong HH, Baek JE, Hong SM, Jeong SY, Koh SS. PAUF as a Target for Treatment of High PAUF-Expressing Ovarian Cancer. Front Pharmacol. 2022 May 6;13:890614. doi: 10.3389/fphar.2022.890614
81	Lu H, Shi C, Liu X, Liang C, Yang C, Wan X, Li L, Liu Y. Identification of ZG16B as a prognostic biomarker in breast cancer. Open Med (Wars). 2020 Nov 25;16(1):1-13. doi: 10.1515/med-2021-0004
82	Liu F, Hu L, Ma Y, Huang B, Xiu Z, Zhang P, Zhou K, Tang X. Increased expression of monoamine oxidase A is
83	Peehl DM, Coram M, Khine H, Reese S, Nolley R, Zhao H. The significance of monoamine oxidase-A expression in high grade prostate cancer. J Urol. 2008 Nov;180(5):2206-11. doi: 10.1016/j.juro.2008.07.019

84	Ponnusamy MP, Seshacharyulu P, Vaz A, Dey P, Batra SK. MUC4 stabilizes HER2 expression and maintains the cancer stem cell population in ovarian cancer cells. J Ovarian Res. 2011 Apr 26;4(1):7. doi: 10.1186/1757-2215-4-7
85	Chauhan SC, Singh AP, Ruiz F, Johansson SL, Jain M, Smith LM, Moniaux N, Batra SK. Aberrant expression of MUC4 in ovarian carcinoma: diagnostic significance alone and in combination with MUC1 and MUC16 (CA125). Mod Pathol. 2006 Oct;19(10):1386-94. doi: 10.1038/modpathol.3800646
86	Kader T, Lin JR, Hug CB, Coy S, Chen YA, de Bruijn I, Shih N, Jung E, Pelletier RJ, Lopez Leon M, Mingo G, Omran DK, Lee JS, Yapp C, Satravada BA, Kundra R, Xu Y, Chan S, Tefft JB, Muhlich JL, Kim SH, Gysler SM, Agudo J, Heath JR, Schultz N, Drescher CW, Sorger PK, Drapkin R, Santagata S. Multimodal Spatial Profiling Reveals Immune Suppression and Microenvironment Remodeling in Fallopian Tube Precursors to High-Grade Serous Ovarian Carcinoma. Cancer Discov. 2024 Dec 20. doi: 10.1158/2159-8290.CD-24-1366
87	Ye Y, Dai Q, Qi H. A novel defined pyroptosis-related gene signature for predicting the prognosis of ovarian cancer. Cell Death Discov. 2021 Apr 7;7(1):71. doi: 10.1038/s41420-021-00451-x
88	Peng L, Zhu N, Wang D, Zhou Y, Liu Y. Comprehensive Analysis of Prognostic Value and Immune Infiltration of NLRC4 and CASP1 in Colorectal Cancer. Int J Gen Med. 2022 Jun 3;15:5425-5440. doi: 10.2147/IJGM.S353380
89	Gao W, Zhang ZW, Wang HY, Li XD, Peng WT, Guan HY, Liao YX, Liu A. TMED2/9/10 Serve as Biomarkers for Poor Prognosis in Head and Neck Squamous Carcinoma. Front Genet. 2022 Jun 8;13:895281. doi: 10.3389/fgene.2022.895281
90	Arentz G, Mittal P, Klingler-Hoffmann M, Condina MR, Ricciardelli C, Lokman NA, Kaur G, Oehler MK, Hoffmann P. Label-Free Quantification Mass Spectrometry Identifies Protein Markers of Chemotherapy Response in High-Grade Serous Ovarian Cancer. Cancers (Basel). 2023 Apr 6;15(7):2172. doi: 10.3390/cancers15072172
91	Yao M, Fu L, Liu X, Zheng D. In-Silico Multi-Omics Analysis of the Functional Significance of Calmodulin 1 in Multiple Cancers. Front Genet. 2022 Jan 12;12:793508. doi: 10.3389/fgene.2021.793508
92	Liu T, Han X, Zheng S, Liu Q, Tuerxun A, Zhang Q, Yang L, Lu X. CALM1 promotes progression and dampens chemosensitivity to EGFR inhibitor in esophageal squamous cell carcinoma. Cancer Cell Int. 2021 Feb 18;21(1):121. doi: 10.1186/s12935-021-01801-6
93	Handley KF, Mehta S, Martin AL, Biswas S, Maharaj K, Nagy MZ, Mine JA, Cortina C, Yu X, Sprenger K, Mandal G, Innamarato P, Powers JJ, Harro CM, Chaurio RA, Anadon CM, Shahzad MM, Flores I, Conejo-Garcia JR. Actionable spontaneous antibody responses antagonize malignant progression in ovarian carcinoma. Gynecol Oncol. 2023 Jun;173:114-121. doi: 10.1016/j.ygyno.2023.03.020
94	Yang Y, Hong Q, Shi P, Liu Z, Luo J, Shao Z. Elevated expression of syntenin in breast cancer is correlated with lymph node metastasis and poor patient survival. Breast Cancer Res. 2013 Jun 20;15(3):R50. doi: 10.1186/bcr3442
95	Iwamoto K, Takahashi H, Okuzaki D, Osawa H, Ogino T, Miyoshi N, Uemura M, Matsuda C, Yamamoto H, Mizushima T, Mori M, Doki Y, Eguchi H. Syntenin-1 promotes colorectal cancer stem cell expansion and chemoresistance by regulating prostaglandin E2 receptor. Br J Cancer. 2020 Sep;123(6):955-964. doi: 10.1038/s41416-020-0965-9
96	Das SK, Maji S, Wechman SL, Bhoopathi P, Pradhan AK, Talukdar S, Sarkar D, Landry J, Guo C, Wang XY, Cavenee WK, Emdad L, Fisher PB. MDA-9/Syntenin (SDCBP): Novel gene and therapeutic target for cancer metastasis. Pharmacol Res. 2020 May;155:104695. doi: 10.1016/j.phrs.2020.104695
97	Puiffe ML, Le Page C, Filali-Mouhim A, Zietarska M, Ouellet V, Tonin PN, Chevrette M, Provencher DM, Mes-Masson AM. Characterization of ovarian cancer ascites on cell invasion, proliferation, spheroid formation, and gene expression in an in vitro model of epithelial ovarian cancer. Neoplasia. 2007 Oct;9(10):820-9. doi: 10.1593/neo.07472
98	Gou R, Zhu L, Zheng M, Guo Q, Hu Y, Li X, Liu J, Lin B. Annexin A8 can serve as potential prognostic biomarker and therapeutic target for ovarian cancer: based on the comprehensive analysis of Annexins. J Transl Med. 2019 Sep 2;17(1):275. doi: 10.1186/s12967-019-2023-z

99	Jiang G, Wang P, Wang W, Li W, Dai L, Chen K. Annexin A13 promotes tumor cell invasion in vitro and is associated with metastasis in human colorectal cancer. Oncotarget. 2017 Mar 28;8(13):21663-21673. doi: 10.18632/oncotarget.15523
100	Shen C, Zhang S, Zhang Z, Yang S, Zhang Y, Lin Y, Fu C, Li Z, Wu Z, Wang Z, Li Z, Guo J, Li P, Hu H. Pan-cancer evidence of prognosis, immune infiltration, and immunotherapy efficacy for annexin family using multi-omics data. Funct Integr Genomics. 2023 Jun 26;23(3):211. doi: 10.1007/s10142-023-01106-z
101	Xiao Q, Qu K, Wang C, Kong Y, Liu C, Jiang D, Saiyin H, Jia F, Ni C, Chen T, Zhang Y, Zhang P, Qin W, Sun Q, Wang H, Yi Q, Liu J, Huang H, Yu L. HDGF-related protein-3 is required for anchorage-independent survival and chemoresistance in hepatocellular carcinomas. Gut. 2013 Mar;62(3):440-51. doi: 10.1136/gutjnl-2011-300781
102	Zhang W, Zhang S, Guan W, Huang Z, Kong J, Huang C, Wang H, Yang S. Poly C Binding Protein 1 Regulates p62/SQSTM1 mRNA Stability and Autophagic Degradation to Repress Tumor Progression. Front Genet. 2020 Aug 14;11:930. doi: 10.3389/fgene.2020.00930
103	Thakur S, Nakamura T, Calin G, Russo A, Tamburrino JF, Shimizu M, Baldassarre G, Battista S, Fusco A, Wassell RP, Dubois G, Alder H, Croce CM. Regulation of BRCA1 transcription by specific single-stranded DNA binding factors. Mol Cell Biol. 2003 Jun;23(11):3774-87. doi: 10.1128/MCB.23.11.3774-3787.2003
104	Guo J, Zhu C, Yang K, Li J, Du N, Zong M, Zhou J, He J. Poly(C)-binding protein 1 mediates drug resistance in colorectal cancer. Oncotarget. 2017 Feb 21;8(8):13312-13319. doi: 10.18632/oncotarget.14516
105	Chen Z, Wang C, Ding J, Yu T, Li N, Ye C. Construction and analysis of competitive endogenous RNA networks and prognostic models associated with ovarian cancer based on the exoRBase database. PLoS One. 2024 Apr 11;19(4):e0291149. doi: 10.1371/journal.pone.0291149
106	Wang H, Zhao LN, Li KZ, Ling R, Li XJ, Wang L. Overexpression of ribosomal protein L15 is associated with cell proliferation in gastric cancer. BMC Cancer. 2006 Apr 11;6:91. doi: 10.1186/1471-2407-6-91
107	Yan TT, Fu XL, Li J, Bian YN, Liu DJ, Hua R, Ren LL, Li CT, Sun YW, Chen HY, Fang JY, Hong J. Downregulation of RPL15 may predict poor survival and associate with tumor progression in pancreatic ductal adenocarcinoma. Oncotarget. 2015 Nov 10;6(35):37028-42. doi: 10.18632/oncotarget.5939
108	He Z, Deng T, Duan X, Zeng G. Profiles of overall survival-related gene expression-based risk signature and their prognostic implications in clear cell renal cell carcinoma. Biosci Rep. 2020 Sep 30;40(9):BSR20200492. doi: 10.1042/BSR20200492
109	Ren X, Liang S, Li Y, Ji Y, Li L, Qin C, Fang K. ENAM gene associated with T classification and inhibits proliferation in renal clear cell carcinoma. Aging (Albany NY). 2021 Feb 3;13(5):7035-7051. doi: 10.18632/aging.202558
110	Xu Q, Kong N, Zhao Y, Wu Q, Wang X, Xun X, Gao P. Pan-Cancer Analyses Reveal Oncogenic and Immunological Role of PLOD2. Front Genet. 2022 May 2;13:864655. doi: 10.3389/fgene.2022.864655
111	Wilken JA, Badri T, Cross S, Raji R, Santin AD, Schwartz P, Branscum AJ, Baron AT, Sakhitab AI, Maihle NJ. EGFR/HER-targeted therapeutics in ovarian cancer. Future Med Chem. 2012 Mar;4(4):447-69. doi: 10.4155/fmc.12.11
112	Gui T, Shen K. The epidermal growth factor receptor as a therapeutic target in epithelial ovarian cancer. Cancer Epidemiol. 2012 Oct;36(5):490-6. doi: 10.1016/j.canep.2012.06.005
113	Li D, Bi FF, Cao JM, Cao C, Li CY, Yang Q. Effect of BRCA1 on epidermal growth factor receptor in ovarian cancer. J Exp Clin Cancer Res. 2013 Dec 9;32(1):102. doi: 10.1186/1756-9966-32-102
114	Yarden Y, Pines G. The ERBB network: at last, cancer therapy meets systems biology. Nat Rev Cancer. 2012 Jul 12;12(8):553-63. doi: 10.1038/nrc3309
115	Luo D, Li X, Wei L, Yu Y, Hazaisihan Y, Tao L, Li S, Jia W. Ubiquitin-related gene markers predict immunotherapy response and prognosis in patients with epithelial ovarian carcinoma. Sci Rep. 2024 Oct 24;14(1):25239. doi: 10.1038/s41598-024-76945-2
116	Li X, Ruan Z, Yang S, Yang Q, Li J, Hu M. Bioinformatic-Experimental Screening Uncovers Multiple Targets for Increase of MHC-I Expression through Activating the Interferon Response in Breast Cancer. Int J Mol Sci. 2024 Sep 30;25(19):10546. doi: 10.3390/ijms251910546

117	Liang X, Hong A, Shen R, Zhu M, Tian W. NCKAP1 as a prognostic and immunological biomarker: pan-cancer analysis and validation in renal clear cell carcinoma. Am J Transl Res. 2024 Aug 15;16(8):4083-4100. doi: 10.62347/UKQB2042
118	Pu J, Ai T, Weng W, Wang L, Yang Y, Ma L, Hu Z, Meng X. TJP1, a Membrane-Expressed Protein, is a Potential Therapeutic and Prognostic Target for Lung Cancer. Technol Cancer Res Treat. 2022 Jan-Dec;21:15330338221106855. doi: 10.1177/15330338221106855
119	Liu XQ, Shao XR, Liu Y, Dong ZX, Chan SH, Shi YY, Chen SN, Qi L, Zhong L, Yu Y, Lv T, Yang PF, Li LY, Wang XB, Zhang XD, Li X, Zhao W, Sehgal L, Li M, Zhang XD. Tight junction protein 1 promotes vasculature remodeling via regulating USP2/TWIST1 in bladder cancer. Oncogene. 2022 Jan;41(4):502-514. doi: 10.1038/s41388-021-02112-w
120	Newtson A, Reyes H, Devor EJ, Goodheart MJ, Bosquet JG. Identification of Novel Fusion Transcripts in High Grade Serous Ovarian Cancer. Int J Mol Sci. 2021 Apr 30;22(9):4791. doi: 10.3390/ijms22094791
121	Zhang J, Li Y, Zou J, Lai CT, Zeng T, Peng J, Zou WD, Cao B, Liu D, Zhu LY, Li H, Li YK. Comprehensive analysis of the glutathione S-transferase Mu (GSTM) gene family in ovarian cancer identifies prognostic and expression significance. Front Oncol. 2022 Jul 28;12:968547. doi: 10.3389/fonc.2022.968547
122	Hao X, Zhang J, Chen G, Cao W, Chen H, Chen S. Aberrant expression of GSTM5 in lung adenocarcinoma is associated with DNA hypermethylation and poor prognosis. BMC Cancer. 2022 Jun 21;22(1):685. doi: 10.1186/s12885-022-09711-0
123	Jou YC, Wang SC, Dia YC, Wang ST, Yu MH, Yang HY, Chen LC, Shen CH, Liu YW. Anti-Cancer Effects and Tumor Marker Role of Glutathione S-Transferase Mu 5 in Human Bladder Cancer. Int J Mol Sci. 2021 Mar 17;22(6):3056. doi: 10.3390/ijms22063056
124	Hua T, Wang RM, Zhang XC, Zhao BB, Fan SB, Liu DX, Wang W. ZNF76 predicts prognosis and response to platinum chemotherapy in human ovarian cancer. Biosci Rep. 2021 Dec 22;41(12):BSR20212026. doi: 10.1042/BSR20212026
125	Xu F, Kong L, Sun X, Hui W, Jiang L, Han W, Xiao Z, Li N, Chen D, Zheng N, Han J, Liu L. PFDN6 contributes to colorectal cancer progression via transcriptional regulation. eGastroenterology. 2024 Apr 10;2(2):e100001. doi: 10.1136/egastro-2023-100001
126	Nikolatou K, Sandilands E, Román-Fernández A, Cumming EM, Freckmann E, Lilla S, Buetow L, McGarry L, Neilson M, Shaw R, Strachan D, Miller C, Huang DT, McNeish IA, Norman JC, Zanivan S, Bryant DM. PTEN deficiency exposes a requirement for an ARF GTPase module for integrin-dependent invasion in ovarian cancer. EMBO J. 2023 Sep 18;42(18):e113987. doi: 10.15252/embj.2023113987
127	Jin KL, Pak JH, Park JY, Choi WH, Lee JY, Kim JH, Nam JH. Expression profile of histone deacetylases 1, 2 and 3 in ovarian cancer tissues. J Gynecol Oncol. 2008 Sep;19(3):185-90. doi: 10.3802/jgo.2008.19.3.185
128	de Heer EC, Zois CE, Bridges E, van der Vegt B, Sheldon H, Veldman WA, Zwager MC, van der Sluis T, Haider S, Morita T, Baba O, Schröder CP, de Jong S, Harris AL, Jalving M. Glycogen synthase 1 targeting reveals a metabolic vulnerability in triple-negative breast cancer. J Exp Clin Cancer Res. 2023 Jun 6;42(1):143. doi: 10.1186/s13046-023-02715-z
129	Dogan B, Gumusoglu E, Ulgen E, Sezerman OU, Gunel T. Integrated bioinformatics analysis of validated and circulating miRNAs in ovarian cancer. Genomics Inform. 2022 Jun;20(2):e20. doi: 10.5808/gi.21067
130	Pathak GP, Shah R, Kennedy BE, Murphy JP, Clements D, Konda P, Giacomantonio M, Xu Z, Schlaepfer IR, Gujar S. RTN4 Knockdown Dysregulates the AKT Pathway, Destabilizes the Cytoskeleton, and Enhances Paclitaxel-Induced Cytotoxicity in Cancers. Mol Ther. 2018 Aug 1;26(8):2019-2033. doi: 10.1016/j.ymthe.2018.05.026
131	Li K, Wang R. Unraveling the causal relationship and potential mechanisms between osteoarthritis and breast cancer: insights from mendelian randomization and bioinformatics analysis. Discov Oncol. 2024 Dec 18;15(1):769. doi: 10.1007/s12672-024-01642-5
132	Larsson P, Pettersson D, Engqvist H, Werner Rönnerman E, Forssell-Aronsson E, Kovács A, Karlsson P, Helou K, Parris TZ. Pan-cancer analysis of genomic and transcriptomic data reveals the prognostic relevance of human proteasome genes in different cancer types. BMC Cancer. 2022 Sep 19;22(1):993. doi: 10.1186/s12885-022-10079-4

133	Yi Z, Yang D, Liao X, Guo F, Wang Y, Wang X. PSME3 induces epithelial-mesenchymal transition with inducing the expression of CSC markers and immunosuppression in breast cancer. Exp Cell Res. 2017 Sep 15;358(2):87-93. doi: 10.1016/j.yexcr.2017.05.017
134	Xu Y, Liu X, Cao J, Wu Y, Jiang Q, Luo B. Rho GTPase-activating protein 1 promotes hepatocellular carcinoma progression via modulation by CircPIP5K1A/MiR-101-3p. Hepatol Res. 2024 Feb;54(2):174-188. doi: 10.1111/hepr.13972
135	Li JP, Liu Y, Yin YH. ARHGAP1 overexpression inhibits proliferation, migration and invasion of C-33A and SiHa cell lines. Onco Targets Ther. 2017 Feb 7;10:691-701. doi: 10.2147/OTT.S112223
136	Cheng Z, Chen Y, Huang H. Identification and Validation of a Novel Prognostic Signature Based on Ferroptosis-Related Genes in Ovarian Cancer. Vaccines (Basel). 2023 Jan 17;11(2):205. doi: 10.3390/vaccines11020205
137	An Y, Dong H, Yan M, Liu C, Hu D, Liu Q, Zhang J, Han X, Li Z, Xu M, Chen L, Zhang Q, Gao C. Pan-Cancer Analysis of ANO6 and Experimental Validation in Metastatic Melanoma. Biochem Genet. 2025 Mar 5. doi: 10.1007/s10528-025-11074-7
138	Tang LH, Dai M, Wang DH. ANO6 is a reliable prognostic biomarker and correlates to macrophage polarization in breast cancer. Medicine (Baltimore). 2023 Nov 10;102(45):e36049. doi: 10.1097/MD.0000000000036049
139	Jiang L, Liu JY, Shi Y, Tang B, He T, Liu JJ, Fan JY, Wu B, Xu XH, Zhao YL, Qian F, Cui YH, Yu PW. MTMR2 promotes invasion and metastasis of gastric cancer via inactivating IFNγ/STAT1 signaling. J Exp Clin Cancer Res. 2019 May 21;38(1):206. doi: 10.1186/s13046-019-1186-z
140	Ma H, Qi G, Han F, Lu W, Peng J, Li R, Yan S, Yuan C, Kong B. HMGB3 promotes PARP inhibitor resistance through interacting with PARP1 in ovarian cancer. Cell Death Dis. 2022 Mar 24;13(3):263. doi: 10.1038/s41419-022-04670-7
141	Ma H, Qi G, Han F, Gai P, Peng J, Kong B. HMGB3 promotes the malignant phenotypes and stemness of epithelial ovarian cancer through the MAPK/ERK signaling pathway. Cell Commun Signal. 2023 Jun 16;21(1):144. doi: 10.1186/s12964-023-01172-7
142	Mukherjee A, Huynh V, Gaines K, Reh WA, Vasquez KM. Targeting the High-Mobility Group Box 3 Protein Sensitizes Chemoresistant Ovarian Cancer Cells to Cisplatin. Cancer Res. 2019 Jul 1;79(13):3185-3191. doi: 10.1158/0008-5472
143	Le Tran N, Wang Y, Bilandzic M, Stephens A, Nie G. Podocalyxin promotes the formation of compact and chemoresistant cancer spheroids in high grade serous carcinoma. Sci Rep. 2024 Mar 30;14(1):7539. doi: 10.1038/s41598-024-57053-7
144	Canals Hernaez D, Hughes MR, Li Y, Mainero Rocca I, Dean P, Brassard J, Bell EM, Samudio I, Mes-Masson AM, Narimatsu Y, Clausen H, Blixt O, Roskelley CD, McNagny KM. Targeting a Tumor-Specific Epitope on Podocalyxin Increases Survival in Human Tumor Preclinical Models. Front Oncol. 2022 May 4;12:856424. doi: 10.3389/fonc.2022.856424
145	Wang KH, Chu SC, Chu TY. Loss of calponin h1 confers anoikis resistance and tumor progression in the development of high-grade serous carcinoma originating from the fallopian tube epithelium. Oncotarget. 2017 May 19;8(37):61133-61145. doi: 10.18632/oncotarget.18024
146	Zhou H, Ke J, Liu C, Zhu M, Xiao B, Wang Q, Hou R, Zheng Y, Wu Y, Zhou X, Chen X, Pan H. Potential prognostic and immunotherapeutic value of calponin 1: A pan-cancer analysis. Front Pharmacol. 2023 Apr 21;14:1184250. doi: 10.3389/fphar.2023.1184250
147	Nizioł M, Zińczuk J, Zaręba K, Guzińska-Ustymowicz K, Pryczynicz A. Immunohistochemical Analysis of the Expression of Adhesion Proteins: TNS1, TNS2 and TNS3 in Correlation with Clinicopathological Parameters in Gastric Cancer. Biomolecules. 2021 Apr 26;11(5):640. doi: 10.3390/biom11050640
148	Duan J, Wang L, Shang L, Yang S, Wu H, Huang Y, Miao Y. miR-152/TNS1 axis inhibits non-small cell lung cancer progression through Akt/mTOR/RhoA pathway. Biosci Rep. 2021 Jan 29;41(1):BSR20201539. doi: 10.1042/BSR20201539
149	Lin YS, Tsai YC, Li CJ, Wei TT, Wang JL, Lin BW, Wu YN, Wu SR, Lin SC, Lin SC. Overexpression of NUDT16L1 sustains proper function of mitochondria and leads to ferroptosis insensitivity in colorectal cancer. Redox Biol. 2024 Nov;77:103358. doi: 10.1016/j.redox.2024.103358
